# Detecting subtle yet fast skeletal muscle contractions with ultrasoft and durable graphene-based cellular materials

**DOI:** 10.1093/nsr/nwab184

**Published:** 2021-10-05

**Authors:** Zijun He, Zheng Qi, Huichao Liu, Kangyan Wang, Leslie Roberts, Jefferson Z Liu, Yilun Liu, Stephen J Wang, Mark J Cook, George P Simon, Ling Qiu, Dan Li

**Affiliations:** Department of Chemical Engineering, The University of Melbourne, Melbourne3010, Australia; Department of Materials Science and Engineering, Monash University, Melbourne3800, Australia; Department of Chemical Engineering, Monash University, Melbourne3800, Australia; State Key Laboratory for Strength and Vibration of Mechanical Structures, School of Aerospace Engineering, Xi’an Jiaotong University, Xi’an 710049, China; Department of Chemical Engineering, The University of Melbourne, Melbourne3010, Australia; Neurophysiology Department, Department of Neurology and Neurological Research, St Vincent's Hospital, Melbourne3065, Australia; Department of Medicine, St. Vincent's Hospital, University of Melbourne, Melbourne3010, Australia; Department of Mechanical Engineering, University of Melbourne, Melbourne3010, Australia; State Key Laboratory for Strength and Vibration of Mechanical Structures, School of Aerospace Engineering, Xi’an Jiaotong University, Xi’an 710049, China; Department of Design, Monash University, Melbourne3145, Australia; School of Design, The Hong Kong Polytechnic University, Hong Kong 999077, China; Department of Medicine, St. Vincent's Hospital, University of Melbourne, Melbourne3010, Australia; Department of Materials Science and Engineering, Monash University, Melbourne3800, Australia; Department of Materials Science and Engineering, Monash University, Melbourne3800, Australia; Shenzhen Geim Graphene Center, Tsinghua-Berkeley Shenzhen Institute, Tsinghua University, Shenzhen518055, China; Department of Chemical Engineering, The University of Melbourne, Melbourne3010, Australia; Department of Materials Science and Engineering, Monash University, Melbourne3800, Australia

**Keywords:** cellular graphene, strain sensors, high-frequency electromechanical property, surface mechanomyography, skeletal muscle activity

## Abstract

Human bodily movements are primarily controlled by the contractions of skeletal muscles. Unlike joint or skeletal movements that are generally performed in the large displacement range, the contractions of the skeletal muscles that underpin these movements are subtle in intensity yet high in frequency. This subtlety of movement makes it a formidable challenge to develop wearable and durable soft materials to electrically monitor such motions with high fidelity for the purpose of, for example, muscle/neuromuscular disease diagnosis. Here we report that an intrinsically fragile ultralow-density graphene-based cellular monolith sandwiched between silicone rubbers can exhibit a highly effective stress and strain transfer mechanism at its interface with the rubber, with a remarkable improvement in stretchability (>100%). In particular, this hybrid also exhibits a highly sensitive, broadband-frequency electrical response (up to 180 Hz) for a wide range of strains. By correlating the mechanical signal of muscle movements obtained from this hybrid material with electromyography, we demonstrate that the strain sensor based on this hybrid material may provide a new, soft and wearable mechanomyography approach for real-time monitoring of complex neuromuscular–skeletal interactions in a broad range of healthcare and human–machine interface applications. This work also provides a new architecture-enabled functional soft material platform for wearable electronics.

## Teaser

Stretchable graphene foam enables a new wearable mechanomyographic strategy for monitoring subtle yet fast skeletal muscular activities.

## INTRODUCTION

Our daily life involves a wide range of physical activities ranging from fine movements such as writing to large, gross movements such as running [1,2]. A variety of advanced sensor technologies have been developed and utilized to assess different bodily movements in disease diagnosis, sports training, rehabilitation and human–machine interfaces, aiming at enabling a healthy, mobile and high-quality lifestyle [2–6]. Skeletal muscles underpin most of these bodily movements. After receiving a minute electric signal from the brain through the neurons, skeletal muscles immediately contract via the simultaneous contractions of numerous muscle fibres, and thus drive joint and skeletal movements [1]. Many diseases such as Parkinson's disease and Myasthenia Gravis can be caused by or lead to some impairments of these neuromuscular–skeletal interactions [7–9]. Using sensors to track the complex physical movements of human bodies and monitor the pattern and health status of skeletal muscle contractions can provide valuable information to clinicians for disease diagnosis. For example, electromyography (EMG, using electrochemical sensors to record the electrical activity of muscle tissues) and mechanomyography (MMG, using accelerometers to detect the mechanical motions of skeletal muscles) are already used clinically for neuromuscular/movement disorder diagnosis [8,10,11]. Real-time, accurate monitoring of the activities of skeletal muscles, including their contractions and the skeletal movements, is also highly valuable for sport and professional performance analysis, human–machine interfacing and soft robotics development [2,12–14]. These emerging applications, together with the increasing demand for remote and personalized healthcare monitoring, require that the sensors be compliant and wearable so that real-time and comprehensive information can be collected from the body without sacrificing the wearers’ comfort and independence.

Soft materials play a key role in wearable electronics. In addition to offering the necessary comfort to the wearers, intrinsically soft and stretchable sensors with their mechanical properties are well matched with soft human skin and are in an excellent position to conform to the skin’s motions and provide high-fidelity monitoring [14,15]. Thus sensors based on soft and mechanically durable materials such as rubber have received increasing attention in recent years. Among the variety of soft wearable sensors, rubber-based resistive strain sensors have been widely exploited for monitoring different types of human physical activities because of their combination of softness, low-cost and easy signal reading and interpretation [5,6,14,16–18]. However, human bodily activities are extremely dynamic and complex and different bodily motions exhibit distinct strain and frequency features. For example, joint movements can induce large skin strain up to 55% [6,19] but with a low frequency (<5 Hz); in contrast, inner skeletal muscle fibre contractions are more subtle, with strains as low as 0.5% of the muscle length [20,21], but their frequency can reach up to 40 Hz [22–24]. To our knowledge, previous research on wearable resistive strain sensors has been primarily centred on monitoring different gross skeletal movements such as gait, posture, locomotion or other voluntary movements [3,5,16,17,19,25–33]; little success has been made in the use of flexible soft materials to assess minute neuromuscular–skeletal interactions and more subtle and particularly high-frequency skeletal muscle fibre contractions, likely limited by the viscoelasticity of polymeric elastomers, which would inevitably result in a relatively slow response or delayed recovery. Therefore, the development of soft, stretchable, mechanically durable yet highly sensitive electroconductive materials for detection of complex, subtle and high-frequency dynamic neuromuscular activities represents a significant challenge.

Recent successful syntheses of ultralight, electroactive cellular materials through hierarchical assembly of nanomaterials have made it possible to create ultrasoft electroactive materials with extraordinary electromechanical properties that are unattainable with traditional polymer-based soft materials. By controlled assembly of rigid, conductive nanoscale building blocks, such as carbon nanotubes, gold nanofibers and graphene, into an ultralight three-dimensional highly porous cellular architecture, soft yet highly sensitive and high-frequency responsive materials have been obtained [34–37]. We have recently demonstrated that ultrasoft, super-compressible graphene-based cellular material (UGCM) can exhibit frequency-independent electromechanical responses up to 2000 Hz and an extremely high piezoresistive sensitivity that allows detection of pressures as low as 0.082 Pa [35]. However, architecture-enabled, polymer-free ultrasoft materials are generally too fragile to be directly applied on the skin for skin deformations detection. The deformability and mechanical durability of the ultralight cellular materials can be drastically improved by infiltrating a polymer into the UGCM to form intertwined networks [28,38–43]; however, the intimate interfacing of the conductive network with polymer leads to the elimination of their unique electromechanical dynamic response capability [41] and the ability for detection of extremely low pressure limits [43,44]. To date, it has remained unclear as to whether this issue can be resolved to allow the design and production of stretchable yet resilient sensors based on such a new class of soft materials, when being incorporated with stretchable, durable rubbery materials, to provide mechanical robustness, whilst also being able to retain their frequency-independent, rapid-response electromechanical properties.

In this work, using UGCM as an architecture-enabled model soft electroactive material, we explore a new strategy to interface ultrasoft cellular materials with a rubber using a lamination process. This is found to be successful, with the density of UGCM playing a critical role in its interfacing with polydimethylsiloxane (PDMS). An extremely low density (≤1.0 mg/cm^3^) of UGCM allows for an effective uniform stress and strain transfer at its interface with the rubber without delamination, providing the UGCM with a remarkable stretchability and durability while retaining broadband-frequency responsiveness. This discovery allows us to use the resultant UGCM-polymer laminate as a model sensing material to explore the potential use of resistive-type strain sensors for comprehensive detection of human physical activities at different anatomic scales, and most importantly, for subtle, high-frequency skeletal muscle activity.

## RESULTS AND DISCUSSION

### Laminating UGCM with PDMS for tear-tolerance and stretchability

**Figure 1.**
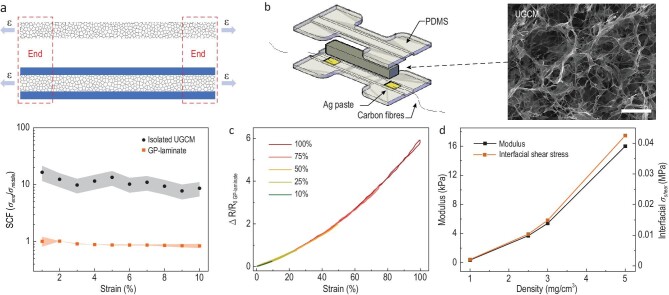
Fabrication of GP-laminate and its mechanical properties. (a) Top: The finite element analysis (FEA) model structure of isolated UGCM and sandwiched UGCM, the loading tensile strain is shown as arrows. Bottom: The computed stress concentration factor (SCF) against the loading tensile strain of isolated UGCM (black curve) and sandwiched UGCM (orange curve). The SCF is defined as the ratio of max principal stresses distributed at the ends and the middle area. The density of the UGCM was 1.0 mg/cm^3^. (b) Schematic diagram of the key construction of the assembled GP-laminate of UGCM with in-built grooves on PDMS layers. A UGCM panel was sandwiched into the groove formed by two PDMS layers. The two ends of the UGCM were separately connected to two soft conductive wires via Ag-pastes. Scale bar: 20 μm. (c) Relative change in resistance of the GP-laminate being stretched to varying levels of strain, indicating its good stretchability and sensing ability. ▵R refers to the resistance changes of the GP-laminate under the specific strain and R_0_ refers to the base resistance of the GP-laminate before stretch. (d) The Young's modulus and the FEA computed mean interfacial shear stress (*σ_shear_*) required as a function of the UGCM density for conformably deforming the GP-laminate under tensile strain.

Lightweight cellular materials are ubiquitous in organisms [45]. Natural cellular materials are generally surrounded by a soft yet denser outer layer to form a hybrid structure able to meet the needs of a dynamic environment [1,45]. Inspired by the manner in which cellular materials are integrated with soft tissue sheaths in biology, the interfacing of the UGCM with PDMS by lamination, rather than infiltration, was designed to improve its stretchability while mitigating the mechanical and electromechanical interference from the polymer by minimizing their contact areas. This strategy can be clearly demonstrated by finite element analysis (FEA) on the deformability of the UGCMs in contact with a PDMS outer layer (see the detailed discussion in the Supplementary Data). We find that when a free-standing UGCM with two ends clamped is stretched, its internal stress is highly concentrated near the clamping area (Fig. 1a, and Figs S1 and S2). This uneven load distribution, together with its highly porous structure, further negatively impacts the fragility and low stretch tolerance of the UGCM [37,46]. In contrast, when the UGCM is in contact with a PDMS

outer layer through lamination, its internal stress distribution can be much more uniformly distributed along the UGCM under stretching (Fig. 1a). This modelling suggests that a possible approach to improving the deformability of UGCM involves encasing it with a soft elastic polymer.

To experimentally examine whether encasing the UGCM with a polymer layer can lead to improved deformability and mechanical durability, we laminate a 1.0 mg/cm^3^ piece of UGCM into a groove formed by two PDMS sheets (Fig. 1b), where the UGCM and the PDMS sheets are physically bonded after the PDMS surface is treated by an oxygen plasma (see detailed discussion in Materials and Methods). Interestingly, we find that bending, twisting or stretching the resultant laminate (denoted as GP-laminate in this work) does not cause visible ruptures in the UGCM (Figs 1c and S3). In stark contrast, slightly stretching a free-standing UGCM (<5% strain) leads to catastrophic breakage. We further monitor the stretchability and connectivity of the UGCM network in the laminate under various stretching conditions by simultaneously recording its electrical resistance, which can increase or decrease with the disconnection or reconnection of the graphene network during the deformations (Figs 1c and S4) [16,47]. The GP-laminate exhibits a nearly linear monotonic and reversible relative resistive change to the externally applied tensile strain up to 100% with negligible hysteresis (Fig. 1c), suggesting a reversible deformation of the UGCM network in the laminate (Fig. S5). Note that a slight deviation of the hysteresis curve of the strain sensor occurred, which could be attributed to the crack generation along the stretching direction and UGCM squeezing in the direction perpendicular to the applied strain.

However, UGCMs with a higher density (e.g. ≥2.5 mg/cm^3^) cannot be made stretchable using this method. When testing these GP-laminates, we observed that the high-density UGCMs were debonded from the PDMS layers and could not be stretched conformably with the PDMS at a strain exceeding ∼5% (Fig. S6). An extremely low density ($\rho $) of UGCM is crucial to the formation of a stretchable laminate. We attribute this phenomenon to the unique mechanical property of the extremely low-density UGCM. Note that when the GP-laminate is stretched, the deformation of the UGCM is driven by the interfacial shear stress transferred from the PDMS layers. A conformable deformation of UGCM (without delamination) should only be possible when the required interfacial stress (*σ_shear_)* to deform the UGCM is lower than its interfacial bonding strength (*σ*_*strength*_) to the PDMS layers. The FEA analysis shows that the *σ_shear_* required to deform a UGCM is proportional to its Young's modulus (*E*), which represents its resistance to the elastic deformation (Fig. 1d). It has been previously reported that the *E* of UGCM follows a scaling relationship with its density as $E \sim {{\rho}^{2}}$ [47]. Therefore, the *σ_shear_* should also follow a relationship of *σ_shear_ ∼*${{\rho}^{2}}$ with the UGCM’s density. On the other hand, owing to the unique porous structure of the UGCM (i.e. UGCM is only physically bonded to the PDMS surface through the limited contact area (Fig. S7)), the *σ_strength_* should be proportional to the wall-PDMS contacting area and thus the UGCM density, i.e. *σ_strength_* ∼ $\rho $ (see detailed discussion in the Supplementary Data). As a lower $\rho $ leads to a greater drop of *σ_shear_* than *σ_strength_*, the UGCM with a lower $\rho $ should be more prone to achieving a conformable stretch (*σ_shear_* <* σ_strength_*), which is consistent with our experimental observations. Our laminate design thus makes it possible to harness the unique scaling relation of *σ_shear_*–$\rho $ and *σ_strength_*–$\rho $ of the UGCM to enable their remarkable stretchability and durability.

### Resistive response of the GP-laminate to broadband strains and frequencies

**Figure 2.**
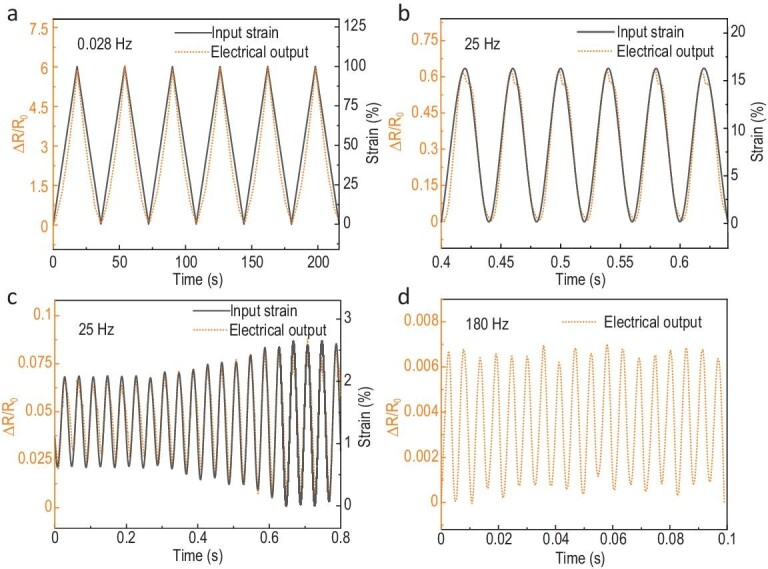
Electromechanical response of GP-laminate to varied frequencies. (a) Relative changes in resistances of the hybrid (orange curve) in response to periodic tensile loading cycles under strains (black curve) of 100%. The cyclic loading frequency was set at 0.028 Hz. (b–d) Very close correspondence observed between the high-frequency dynamic behaviours of the GP-laminate. Relative changes in resistance of the GP-laminate are shown in response to different periodic tensile loading cycles under varied strains with loading frequencies at 25 Hz (b and c) and 180 Hz (d). Note that the cyclic deformation of the applied strain input at 180 Hz was not shown because such a high-frequency strain input was beyond the limit of the laser detector we used.

We have previously demonstrated that free-standing UGCMs exhibit a frequency-independent piezoresistive pressure response owing to their combination of high conductivity and rapid stress transmission rate throughout the entire UGCM network, which far surpasses that of the existing conductive soft polymeric elastomers [35]. Given that the contact between the UGCM and polymer in the GP-laminate is restricted at their external interface only, it is possible that there may be little effect on the electromechanical properties of the laminate. To study the dynamic electromechanical property of the GP-laminate, the *in-situ* electrical characterization is coupled to the periodic sinusoidal tensile mechanical deformations. A monotonic, almost linear change of the electrical response of the laminate to the applied cyclic deformation from subtle strains up to 100% was observed (Figs 2a and S8). Besides, the GP-laminate was able to maintain its reversible deformability by presenting a stable electrical resistance change during the cyclic deformations for at least 1000 cycles (Fig. S9).

The GP-laminate exhibits a nearly instantaneous and linear response to the applied strains at all the tested frequencies up to 180 Hz (the highest limit of the testing equipment) (Fig. 2). The maximal signal delay of the GP-laminate to the input strain is measured to be <13 ms in the strain ranges tested, and further reduced to ∼3 ms at higher frequency (∼25 Hz). Previous studies have shown that rubber-infiltrated conductive composites in which the graphene conductive network was entirely embedded in the rubber matrix displayed a slow electrical signal response during deformation [26,35], even if they had demonstrated the resistive response of a graphene-rubber composite at high frequencies up to 160 Hz. Fully infiltrating the entire graphene network with a viscoelastic polymer resulted in the elimination of the intrinsic electromechanical properties of UGCM because the conductive networks were entangled strongly with the polymer chains and disturbed significantly by the rearrangement of the polymeric chains during deformations. In contrast, the interactions of graphene and PDMS only occurred at their external surface in the GP-laminate; a polymer-free conductive network pathway of graphene is thus maintained in the hybrid. Hence, their electrical disconnection–reconnection is barely influenced by the rearrangement of the internal polymer chains and their electromechanical response is decoupled from the viscoelastic behaviour of the PDMS during the deformations.

### GP-laminate for resistive detection of skeletal muscle contraction

**Figure 3.**
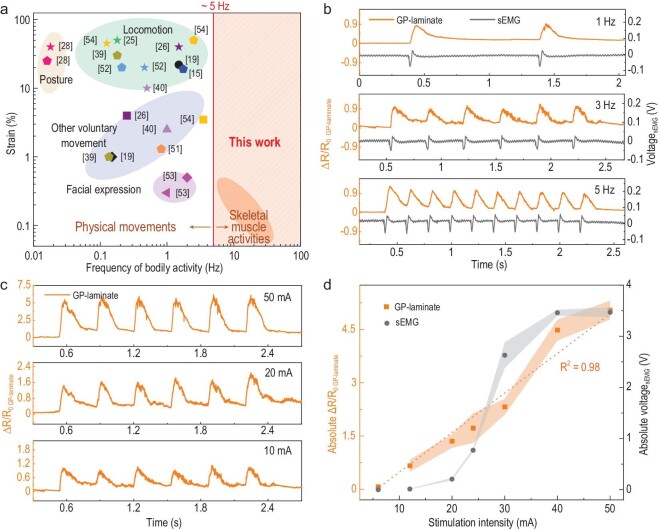
Monitoring of electrically evoked bicep muscle contractions by GP-laminate and sEMG. (a) An illustration of different skeletal-muscle-contraction-related bodily activities located at different strain and frequency ranges, including inner skeletal muscle activities and their manipulated physical activities. Only several representative strain sensors reported in monitoring human bodily activity are presented here for the clarity of this graph [15,19,25,26,28,39,40,51–54]. (b) A comparison of the recorded relative resistance changes of the GP-laminate (orange curve) and voltage outputs of the sEMG (black curve), referring to the muscle action potential, in response to the evoked bicep muscle contractions at different frequencies of 1 Hz (top), 3 Hz (middle) and 5 Hz (bottom). (c) The relative resistance changes of the GP-laminate in response to the evoked bicep muscle contractions when the stimulation intensity was adjusted at 50 mA (top), 20 mA (middle) and 10 mA (bottom). The stimulation frequency was set at 3 Hz. (d) A comparison of peak-to-trough values of the relative resistance changes of the GP-laminate (orange dots), denoted as the ‘absolute ▵R/R_0 GP-laminate_’, with the peak-to-peak range changes of the recorded muscle action potential from the sEMG (black dots), denoted as the ‘absolute Voltage _sEMG_’_,_ in response to the evoked bicep muscle contractions at different stimulation intensities from 6 mA to 50 mA. The variation in these two signal outputs, indicated as error bars, were calculated from nine test results. The GP-laminate signals were recorded by three GP-laminate sensors with similar gauge factors.

The unique electromechanical properties of GP-laminate, together with its great flexibility, mechanical durability and similar mechanical compliance to human skin (Fig. S10), make it promising for use as a skin-mountable sensor for human bodily activity detection and tests in these areas were undertaken. We first tested typical bodily physical movements with relatively large strains whose frequency ranges are generally <5 Hz. As with other existing strain sensors [28,40,41,48–50], GP-laminates can monitor hand and forearm movements from the skin at both slow- and fast-moving tempos (Figs S11 and S12). However, to the best of our knowledge, no reports have demonstrated the use of resistive strain sensors to monitor the more subtle and high-frequency skeletal muscle activities that underpin those bodily movements (Fig. 3a) [15,19,25,26,28,39,40,51–54], hence we focus here on using the GP-laminates as a model material to explore this aspect.

The contraction of skeletal muscles can be evoked voluntarily or through an external electrical stimulation. In this work, GP-laminates were used to monitor bicep muscle contractions evoked by a clinical electrical stimulator with well-controlled stimulation frequencies and intensities (see detailed discussion in the Supplementary Data). Instead of attaching the GP-laminate to the skin near the joint/wrist areas, we place it right onto the belly area of the bicep muscle group and band around the arm to record the relative resistance change of the GP-laminate during muscle contractions (Fig. S13, and Fig. 3b and c). For all the given stimulation frequencies of 1, 3 and 5 Hz, the GP-laminate responds well to the sEMG in the corresponding frequency ranges (Fig. 3b), and the response is nearly linear to the evoked muscle contraction within the stimulation intensity ranges examined (Fig. 3c and d). We used the widely used surface electromyography (sEMG) to simultaneously monitor the electrical aspect of the skeletal muscle activities. The resistive signal pulses recorded from the GP-laminate were in good agreement with the sEMG recordings from both the frequency and relative intensity aspects, with the exception that a slight signal latency of around 1–7 ms between the GP-laminate and the sEMG recordings was observed (Figs S14 and S15). The GP-laminate was also found to be responsive to the other skeletal muscle groups we examined, such as the wrist flexor muscle, and the results also resonated well with the sEMG signal (Fig. S16).

**Figure 4.**
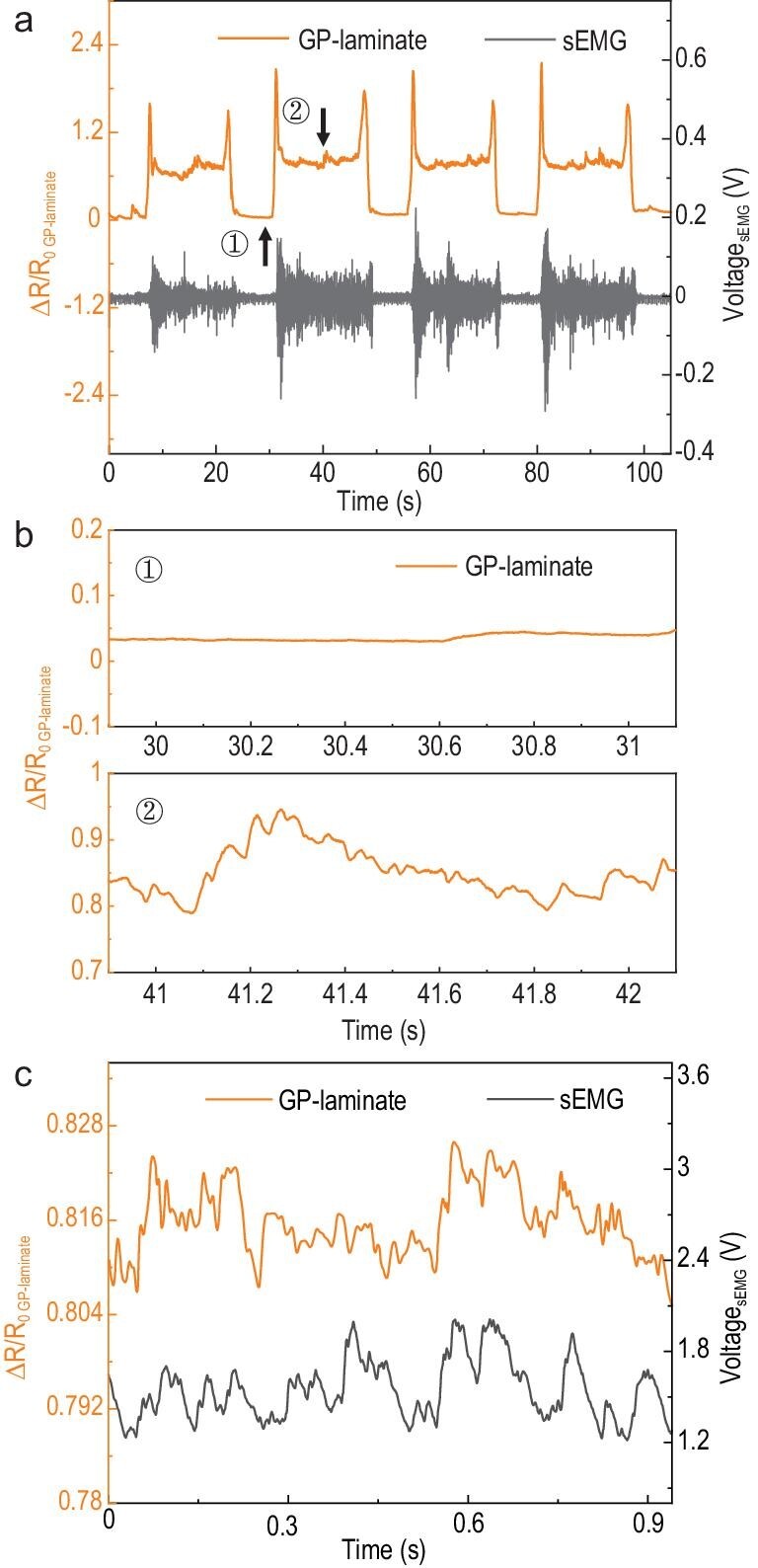
Monitoring of voluntary bicep muscle contractions by the GP-laminates. (a) The recorded resistance changes of the GP-laminate (orange curve) and the muscle action potential extracted by the sEMG (black curve) in response to the multiple recurring voluntary relaxing/contracting cycles of the target bicep muscle group. (b) The recorded resistance changes of the GP-laminate under muscle relaxation (top, section 1) and muscle contraction (bottom, section 2) during the cyclic voluntary muscle contraction detection. (c) A comparison of the resistance changes recording of the GP-laminate (orange curve) and the processed sEMG signal via an in-built data process system of the sEMG (black curve), EMG’s linear envelope, for detecting the recurrent muscle contractions within a 1 s period of time.

We further tested the capability of the GP-laminates with regard to monitoring voluntary muscle contractions. Voluntary muscle contractions can be treated as the summation of single motor-unit twitches or contractions that are artificially produced by recurrent nerve stimulations [23]. Here we adopt a widely studied bicep muscle group as an example in which the GP-laminate was used to monitor voluntary bicep muscle contractions. We found that during multiple relaxation and contraction cycles, both the GP-laminate and sEMG generate an instant response to the voluntary bicep muscle contractions with similar contracting periods and ranges (Fig. 4a). During muscle relaxation, the GP-laminates illustrate a nearly unchanged relative resistance variation of ∼0.03. In contrast, when the bicep muscle is contracted, overall relative resistance changes of the GP-laminate increase to around 0.8 and fluctuate from 0.79 to 0.95 (Fig. 4b). These distinctive sub-peaks are located in the frequency range at around 15 Hz. The output signal from the GP-laminate at this relatively higher frequency range was compared with the simultaneously recorded sEMG signal. The sEMG signal was processed via a standard data processing technique (sEMG linear envelope) to allow interpretation of the mechanical activity of muscle fibres from the EMG signal [55]. It can be observed that the GP-laminate demonstrates similar major waveform patterns to that of the processed sEMG signal (Fig. 4c).

### Using GP-laminate for muscle mechanomyogram

Human skeletal movements are driven and controlled by the contractions of skeletal muscles connected to the joints. By monitoring the deformation of skeletal muscle groups, different types of skeletal movements such as gait or hand postures can be recognized, analysed and controlled by a number of flexible sleeve-based sensor systems [4,56–59]. However, human bodily activities are complex as these activities are not only the behaviours that we can easily notice such as simple joint movements or muscle shape changes but also involve the neuromuscular-skeletal event that drives these joint movements [1,2]. Specifically, a contraction of the skeletal muscle occurs as a result of an electromechanical coupling induced by neuromuscular-skeletal interactions: the electrical and chemical signals generated by the brain pass along the neuron and reach the presynaptic membrane and then stimulate the release of acetyl choline at the synapse. This then crosses the synaptic cleft to the receptors and stimulates the muscle fibres’ action potential change, which causes their contraction [1]. Any abnormality or interruption of the signal propagation through these events can cause muscle dysfunction and impaired movements. To quantitatively examine the complex neuromuscular-skeletal interactions for muscle/neuromuscular disease diagnosis and real-time health monitoring, and for prosthetic and soft robotic development, complementary sensing techniques are often required to accurately collect all electrical and mechanical muscle activities. So far, both EMG and MMG have been developed to collect the electrical and mechanical signals of skeletal muscles, respectively [1,60]. While several types of EMG that can be integrated in epidermal/implantable electronics have been adopted clinically [12,61], the development of MMG devices is much more challenging due to the great diversity and complexity of mechanical activities associated with the contraction of skeletal muscles. Although a range of transducers based on condenser/contact microphones [62,63], piezoelectric contact sensors [64], accelerometers [65,66] and force-resistive sensors [55,67,68] have been used for MMG, existing MMG systems are generally bulky and rigid. The design of fast responsive MMG sensors based on wearable soft materials that can complement wearable EMG devices for comprehensive analysis of dynamic muscle activity has been particularly challenging. This is beneficial to different medical conditions ranging from neurological study to sports analysis and rehabilitation.

Our correlation analysis on the resistive signals obtained from the GP-laminate sensor with corresponding inner mechanical activities of skeletal muscles suggest that our material may provide a new soft, wearable and stretchable MMG device able to monitor the mechanical response of skeletal muscles. It has previously been documented that the volume of skeletal muscles remains nearly constant during contractions [69]. As a result, the shortening of muscle fibres during contractions will cause transversal expansion of the muscle group and lead to a circumferential increase of the skin over the muscle. We have found that when banded around the target muscle group during exercise, the GP-laminate exhibits distinct resistance responses to muscle contractions during the entire process (Fig. 3b and c, and Fig. 4a): at the onset of contraction (which can be timed by EMG), the GP-laminate shows a rapid increase of its resistance change corresponding to the circumferential expansion of the arm; the resistance then drops immediately back to its original value when the muscle starts to relax. The resistance change of the GP-laminate for muscle contraction measurement showed an almost linear response to the applied muscle contraction intensity (Fig. 3d). We also observed a few sharp resistance peaks in the GP-laminate's signal at the onsets and offsets of muscle contractions, which can be attributed to the sudden shape changes of the muscle group (Fig. 4a). This is because muscle groups are not perfectly cylindrically symmetric and contain both contractile tissues and non-contractile elements (i.e. tendons, various sheaths, etc.) that deform differently during muscle contractions, which will cause the lateral movements and changes in shape of the muscle when a sudden contraction or relaxation occurs [1,10,70]. We also observe a very small decrease in the resistance of the GP-laminate just before its strong resistance increase when muscle contractions are electrically evoked (Fig. S14). This is in agreement with a previous finding that, when being electrically stimulated, the skeletal muscle fibre tends to show a minute relaxation (muscle fibre lengthening) before the contraction starts [71–73]. Moreover, during muscle contractions, the active muscle fibres can generate vibrations up to 40 Hz due to their cyclic twitches and shortening depending on the contraction force exertion [74,75]. The detected sub-peaks from the resistive signal of the GP-laminate are found to fall in the same frequency range (Fig. 4b and c). All of these results confirm that the GP-laminate-based strain sensor is indeed able to detect various minute mechanical movements of the skeletal muscles.

It is worth noting that because neuron transition and muscle contractions occur consecutively, there is always a short time delay (generally several milliseconds) between electrical muscle activation and mechanical muscle contractions, the so-called ‘electromechanical latency’ (EML) [72,76,77]. Accurate detection of the EML is crucial to understanding dynamic neuromuscular-skeletal events, particularly for the development of high-precision prosthetics. In this regard, the GP-laminate based MMG sensor seems to be particularly attractive owing to its fast electromechanical response. We have found that the EML detected by our sensor is between ∼1 to 7.6 ms (Fig. S15). This value surpasses that of most reported MMG transducers for the detection of the same bicep muscle group contraction (up to 37 ms) [77]. To our knowledge, such fast dynamic muscle fibre activities have been either non-detectable or have not yet been demonstrated by traditional stretchable conductive rubbers [57–59], likely due to the frequency-dependent viscoelastic property of the polymeric matrix. Such broadband sensing of skeletal muscle activities by using the GP-laminate holds great potential to extend the application scope of soft wearable sensors and unveil new possible opportunities for detecting more complicated and practical neuromuscular events, such as muscle fatigue detection, force exertion assessment or intelligent muscle development, which in turn, can also help to control precise prosthetic movement or drug delivery in response to the feedback loop provided by the soft wearable platforms.

## CONCLUSION

In conclusion, we have demonstrated that ultralight graphene-based cellular materials exhibit an unusual interface stress and strain transfer mechanism when they are laminated with PDMS due to their extremely low density. This has made it possible to make intrinsically non-stretchable and mechanically fragile UGCMs stretchable and durable whilst still retaining their fast electromechanical properties through lamination. The discoveries have allowed us to use the resultant laminate as a resistive strain sensor to explore for the first time its applications in subtle yet highly dynamic skeletal muscle contraction detection, and explore a new soft and wearable mechanomyography solution for real-time monitoring of the mechanical response of skeletal muscles during their contractions.

We have shown that the coupling of this wearable mechanomyography with electromyography can unveil rich real-time information about dynamic neuromuscular-skeletal interactions. Given the critical role of skeletal muscles in human bodily movements, further developing this technique, whilst engaging with advanced data analytics techniques, could be useful for muscle/neuromuscular disease diagnosis, personalized healthcare and the development of prosthetics and soft robotics. The concept revealed here could also inspire the exploration of other architecture-based soft functional materials with regard to making them useable in a mechanically-robust fashion, as well as boost wearable electronics development into tackling more challenging sensor applications and biomedical problems that traditional soft materials are unable to address.

## METHODS

### Fabrication and structural characterization of UGCMs

The UGCMs were fabricated via a modified process of a previously-reported freeze-casting method of partially reduced GO [47]. The typical procedure to synthesize UGCM with a density of 1.0 mg/cm^3^ is as follows: a GO dispersion (12 ml, 1.0 mg/ml) was first mixed with ascorbic acid (AA) at a weight ratio of 4:1 (GO:AA) in a cylindrical glass tube. The mixture was then placed in a boiling water bath for 30 min to obtain a partially reduced graphene hydrogel. The glass tube was subsequently placed in dry ice bath for 45 min to fully freeze the mixture, followed by a freeze-drying process of 2 days. The resultant UGCMs were then annealed at 200°C in air to further reduce the graphene oxide. The UGCMs obtained were then laser cut into cuboids with different dimensions of 12×12×24 mm^3^, 8×8×24 mm^3^ and 6×6×24 mm^3^ for further use. The dimensions and weights of UGCMs were determined with a Vernier calliper (Stamvick) with an accuracy of 0.01 mm and a microbalance (AND, GH-252), with an accuracy of 0.01 mg.

### Fabrication of GP-laminates and GP-laminate-based strain sensors

A typical fabrication process is illustrated in Fig. S17. 3D-printed moulds with a dog-bone shape were first fabricated to produce thin PDMS films with in-depth thicknesses of 100 μm, 200 μm and 500 μm, respectively.

To help maintain the 3D porous structure of UGCMs within the assembling structure, the moulds with an embossment (height, 200 μm) in the middle area of the mould were also prepared. In a typical fabrication process, a mixture containing the silicone elastomer base (Sylgard 184) and the silicone elastomer curing agent (Sylgard 184) (weight ratio at 10:1) was first placed into a vacuum chamber for degassing for 5 min. The degassed PDMS mixture was then put into the mould for curing in air at 50°C for 12 h. The detailed parameters of the substrate design for mechanical tensile tests and for strain sensor assembling of GP-laminates are shown in Figs S18 and S19.

To assemble UGCMs into a GP-laminate, two conductive wires were adhered to the two ends of the PDMS layers with Ag pastes (DuPont Solamet, PV412). The laser-cut UGCMs were then sandwiched into the grooves formed by two PDMS films with the two ends separately connected to the Ag electrode areas. The adhesion at the interfaces of the PDMS and UGCMs was achieved via an oxygen plasma treatment at the PDMS surfaces for 2 min (Harrick Plasma, PDC-002, power 200 W) before assembling. Several critical design parameters, including the thickness of the PDMS layer, the thickness of the groove, the thickness of UGCMs, the density of UGCMs and the interfacial interactions of the assembled GP-laminates were investigated with a comprehensive focus on their resulting stretchability and electromechanical properties (Figs S6, S7, S20 and S21).

## ETHICS DECLARATION

We confirm that we have read the journal's position on issues involved in ethical publication and confirm that this report is consistent with those guidelines. This study received quality-assurance approval by St. Vincent's Hospital Human Research Ethics Committee.

## Supplementary Material

nwab184_Supplemental_File
