# Supplementary material for: Detecting subtle yet fast skeletal muscle contractions with ultrasoft and durable graphene-based cellular materials
Source: Natl Sci Rev. 2021 Oct 5;9(4):nwab184. doi: 10.1093/nsr/nwab184 (PMC8986457; doi:10.1093/nsr/nwab184)
Supplement: nwab184_Supplemental_File [file nwab184_supplemental_file.docx]

**Supplementary Information for**

Detecting subtle yet fast skeletal muscle contractions with ultrasoft and durable graphene-based cellular materials

*Zijun He^1,2^, Zheng Qi^3^, Huichao Liu^4^, Kangyan Wang^1^, Leslie Roberts^5,6^, Jefferson Z. Liu^7^, Yilun Liu^4^, Stephen J. Wang^8,9^, Mark J. Cook^6^, George P. Simon^2^, Ling Qiu^2,10^* and Dan Li^1,2^**

Corresponding author: Dan Li.

Email: [dan.li1@unimelb.edu.au](mailto:dan.li1@unimelb.edu.au).

Corresponding author: Ling Qiu.

Email: [ling.qiu@sz.tsinghua.edu.cn](mailto:ling.qiu@sz.tsinghua.edu.cn).

**This PDF file includes:**

Supplementary text

Fig.s S1 to S22

Tables S1 to S3

SI References 1 to 37

Supplementary text

**Mechanical property characterisations.** The mechanical tensile tests were performed in a mini-Instron (Micro Tester, 5845, Instron) using a 100 N load cell with the strain control mode for low-frequency (< 1 Hz) tensile testing. A small pre-strain (nearly at 2 %) was applied to each sample to ensure no bending of the sample occurred during cyclic deformation. The strain rate was set at 0.5 mm/s and the strain applied was varied from 10% to 100%.

For high-frequency dynamic testing (> 1 Hz), as can be seen in Fig. S18, the tensile tests were performed using an electromagnetic shaker (Bruel & Kjaer, V200), a device that can provide accurate, high-frequency vibrations to produce high-frequency uniaxial deformations on the GP-laminate with adjustable amplitudes. A function generator with a voltage amplifier was used to allow the shaker to generate vibrations with frequencies up to 180 Hz. The strain deformations applied to the GP-laminate were detected by a laser detector (Keyence, LK-G32) which detected the up-down movements of the shaker. Due to the limitation of the sampling rate of the laser, cyclic deformation detections of the shaker were limited to be less than 25 Hz. The setup was placed on a vibration isolation table to minimise environmental vibrations.

**Electromechanical property characterisations.** During electromechanical tests, for low-frequency tensile testing, the electrical response of the hybrid to the applied strain was monitored by a potentiostat (EDAQ, ER466). The sampling rate was set at 1000 s^-1^. During the cyclic stretching of the GP-laminate, a chronoamperometry test mode was used which provided a constant potential of 1.0 V and recorded the current change of the GP-laminate accordingly via two electrodes connected to the two conductive wires at the ends of the GP-laminate.

For the high-frequency tensile tests, as shown in Fig. S19, the strain sensor was connected at both ends in a read-out circuit with an inverted op-amp configuration^1^. The circuit consisted of a surface mounted resistor (R _SMD_), which was fixed at 1 kΩ, and the GP-laminate which acted as another resistor, with a base resistance of ca. 1.1 kΩ. The change in the resistance of the tested GP-laminate was measured and recorded by means of voltage variations for the corresponding strains, and can be derived using Equation S1,

$V strain sensor=- V input \left( \frac{R GP-laminate}{R Resistor} \right)$  **(1)**

where V input is the supply voltage, R _GP-laminate_ and R _Resistor_ refer to the resistance of the tested GP-laminate and the surface mount resistor, respectively. The changes in the R_GP-laminate_ can be calculated from monitoring of the voltage change of the GP-laminates, which was monitored and recorded using a data acquisition card (NI PCI 6251) and LabView for a 2000 Hz sampling frequency. For all the samples, we carried out a five-cycle pre-stretching procedure to stabilise the materials’ structure and electrical signal changes for easier electromechanical characterisation, comparison and analysation. To examine the reproducibility of the electromechanical properties of our GP-laminates, we carried out each electromechanical test by using at least two GP-laminates with the similar structural design.

**Experimental and equipment setup for monitoring skeletal muscle activities.** All tests were performed with a target temperature of the upper limb to be 32 Cº. If required, the limbs were warmed with a heat pack. sEMG was used as the reference to validate the sensing performance of the GP-laminates for skeletal muscle activity detection. Informed consent was provided by all study subjects. The study was approved by the St Vincent’s Hospital Research Ethics Committee and was conducted in accordance with the 1975 Helsinki Declaration. All the equipment was grounded before use. The GP-laminates and sEMG electrodes were placed on the muscle group being studied, after thoroughly cleaning the skin. To avoid the direct contact between the human skin and the conductive wires from the GP-laminate, an insulating and non-elastic band was attached and covered onto the conductive wires that connected to the two ends of the sensor. The GP-laminate was placed in between the reference and active electrodes of the sEMG over the midportion of the muscle under study. The reference electrode was placed on the non-adjacent tissue near the target muscle group. The active electrode was placed as near as possible to the motor point. The electrical signal of GP-laminate signal and the raw sEMG were recorded synchronously by a computerised acquisition system with the assistance of a PowerLab 2/26 and a digital potentiostat 466 System with a sampling rate set at 2000 s^-1^. Two types of sEMG, including the Bio Amp FE231 and a MyowareTM Sensor AT-04-00, were used here for different purposes: the three-lead sEMG (Bio Amp FE231, Signal channel sEMG with a PowerLab 2/26) was used as the primary sensor for detecting electrically evoked muscle activities (Figs 3b and 3d); an alternative sEMG (MyowareTM Sensor AT-04-001) with an in-built rectified-integrated (AKA the EMG’s envelope) was used for voluntary cyclic muscle contraction detections for easier signal analysations and comparison (Fig. 4).

**Detection of electrical stimulated muscle contractions.** Muscle contraction can be evoked by either voluntary or electrical stimulation. To provide predictable muscle contraction behaviours, the biceps muscle was firstly evoked by an electrical stimulator (Dantec Keypoint G4 Workstation, EMG machine) with standard electrodes as used for routine nerve conduction studies. Controlled electrical stimuli produced a peripheral nerve axon action potential that triggers the target muscle’s contractions. The GP-laminate was fixed with two non-elastic soft bands at the two ends with the adhesive rubber pastes and banded onto the arm (Fig. S13). The GP-laminate was placed over the belly of the biceps muscle group as near as possible to the motor point, to optimise the signals that could be recorded from the skin. The GP-laminate was tightened up on the arm, with the application of a small pre-strain (~ 1%). The GP-laminate measured the circumferential changes of the arm. The study was also repeated, recording over the wrist flexor muscle group.

The electrical stimulation pulses were set at 1 Hz, 3 Hz, and 5 Hz, for a period of 2 seconds. For each test, a 5-minute rest was given to allow muscle relaxation. Higher stimulation frequencies (> 5 Hz) were not performed as they may cause strong pain to the subjects. Thus, evaluation of the GP-laminate at higher frequencies was not attempted. Additionally, even under the lower frequency range, the experiment was ceased if the subject felt uncomfortable.

The supramaximal electrical stimulation intensity (defined as the stimulus intensity required to produce a maximal compound muscle action potential) was first determined by a qualified neurologist using the electrical stimulator. To adjust different applied stimulation intensities, the input stimulation current was changed. If required, the duration of the square wave stimulus was increased from 0.1 ms to 0.2 ms. The GP-laminate and sEMG (Bio Amp FE231) signals were recorded synchronously during the tests.

**Detection of voluntary muscle contractions.** The voluntary biceps muscle activities were also monitored by banding the GP-laminate around the arm right onto the belly area. Using a similar protocol to the reference^2^, the subject wore the sensors and sat on a chair with a 90° anteflexion between the arm and the forearm. A weight was hung on the wrist via a soft belt. Cyclic muscle contraction and relaxation were performed. During the muscle contractions, the subject was asked to hold the weight and keep the forearm/arm angle at 90°, with the palm turned towards the shoulder, with the intention being that there was to be no bending at the wrist. This body position can largely minimise the error in measurement caused by the contraction of the adjacent muscle groups (*i.e.,* triceps muscle group) which can also alter the arm circumference changes during contractions. The subject then produced a sustained contraction of the biceps muscle at different forces at 20% MVC, 40 % MVC, 60 % MVC and 80 % MVC for several seconds, respectively. After each test, 1-minute of muscle relaxation was allowed.

During the cyclic voluntary muscle contraction detection (Fig. 4), the MyowareTM Sensor (AT-04-001) was used and to detect the muscle activities synchronously with the GP-laminate. The sEMG is designed to be used directly with a microcontroller which can provide either the primary raw output EMG signal and an alternative amplified, rectified, and integrated EMG signal output (namely EMG linear envelope). The processed the EMG signal is commonly used to interpret the muscle mechanical activity from the EMG signal for easier comparisons to MMG signals^3^. However, the complex data processing procedure could cause delay and show varied signal latency between sEMG and GP-laminates, measured as 15 ms.

**Mechanical investigation based on the Finite Element Analysis (FEA).** This supplementary FEA is focused on the investigation upon the two questions: 1) How can the PDMS enhance the stretchability of the UGCM within the GP-laminate? and 2) how can the density of UGCM impair the stretchability of the GP-laminate?

***Geometry and material models of UGCM and PDMS***

1. Morphology and density of UGCM

UGCM has complex 3D structure^4^ (Fig. S24a). Experimental characterisation has suggested that the UGCM is composed of multi-domains with each domain appearing as a honeycomb-like structure^4^, i.e., hexagons in the cross-section and long channels in the perpendicular direction. Different domains have different orientations in the UGCM. Here, we modelled one domain as a honeycomb with the cross-section as a 2D Voronoi polygons (Fig. S24b), and thus is named as a Voronoi polygon model in the following section. The 3D structure of UGCM is modelled as a random polycrystal (RPC), in which our Voronoi polygon model is a representative single crystal (RSC) domain with transversely isotropic elasticity.

To describe this structure quantitatively, the diameter of cells $d_{c}$ is introduced as the overall control of cell size in Voronoi polygons, which was, for example, about $40\mu m$ for UGCM with density $\rho_{\mathrm{GN}}$= 1.1 mg/cm^3^. The thickness of cell walls $t_{c}$ was determined to be around $4 nm$ for the same specimen^4^. The net volume of cell walls was measured with the help of ABAQUS-CAE. The density of cell walls $\rho_{c}=$ ~ 4 g/cm^3^ can be calculated if given the $\rho_{\mathrm{GN}}$ as 1.1 mg/cm^3^. The morphology of the specimen with the density of 1.1 mg/cm^3^ was regarded as the reference. To model UGCM with other densities of 1.0 mg/cm^3^, 2.5 mg/cm^3^, 3 mg/cm^3^ and 5 mg/cm^3^, the Voronoi polygon morphology and cell wall density $\rho_{c}$ is kept constant and the cell wall thickness $t_{c}$ is changed in order to obtain the target UGCM density $\rho_{\mathrm{GN}}$.

1. The elasticity of the UGCM’s cell wall

The elasticity of cell walls is hard to measure experimentally. The assumption is made that it is an isotopically and linearly elastic material. Specific FEA calculations were employed to determine necessary elastic parameters. Considering the extremely large porosity of UGCM and deformation modes of cell walls, which is mainly stretching and in-plane bending, the influence of Poisson’s effect of the cell walls during the deformation of the whole Voronoi polygon model could be small. Thus, we simply assigned a Poisson’s ratio $\nu_{c}=0.3$ to the cell walls throughout our FEA modelling. The only undetermined elastic parameter was the Young’s modulus of the cell walls $E_{c}$.

We performed a series of FEA modelling of the 2D Voronoi polygon model, including a plane strain stretch test and a pure shear test, with a set of $E_{c}$ values. These FEA calculation determined the elastic parameters of an RSC at the given $E_{c}$. Computational details are presented in the following. Based on the calculated elastic parameters of RSC, the Young’s modulus of corresponding RPC is calculated by the self-consistent method^5-7^. By matching the calculated Young’s modulus of RPC with the experimental results, $E_{c}$ could be determined. The obtained Young’s moduli of cell walls in the relationship to the changed density of UGCM are listed on Table S1. It was found that $E_{c}$ is smaller for a thicker cell wall and may be attributed to the increasing defect density in the thickened cell walls.

FEA is performed with ABAQUS-Explicit-2018, where the Voronoi polygon model has the morphology shown in Fig. S24b with the size of $1.2\times0.4\times0.005$ mm^3^. and overall cell diameter $d_{c}=40 \mu m$. There were about 300 cells. This model was discretised by the structured 4-edge shell elements (S4R) and the controlled global seed of $0.005 mm$.

To extract the elastic parameters, we considered the constitutive relationship of the transversely isotropic materials in terms of compliance matrix (the isotropic plane is parallel with x-o-y plane).

$\left\{ \begin{matrix} \begin{matrix} \varepsilon_{x} \\ \varepsilon_{y} \end{matrix} \\ \begin{matrix} \varepsilon_{z} \\ \gamma_{yz} \end{matrix} \\ \begin{matrix} \gamma_{xz} \\ \gamma_{xy} \end{matrix} \end{matrix} \right\}\boldsymbol{=}\left[ \begin{matrix} \frac{1}{E_{s}} & -\frac{\nu_{s}}{E_{s}} & -\frac{\nu_{ns}}{E_{n}} & & & \\ -\frac{\nu_{s}}{E_{s}} & \frac{1}{E_{s}} & -\frac{\nu_{ns}}{E_{n}} & & & \\ -\frac{\nu_{sn}}{E_{s}} & -\frac{\nu_{sn}}{E_{s}} & \frac{1}{E_{n}} & & & \\ & & & \frac{1}{G_{ns}} & & \\ & & & & \frac{1}{G_{ns}} & \\ & & & & & \frac{2\left( 1+\nu_{s} \right)}{E_{s}} \end{matrix} \right]\left\{ \begin{matrix} \begin{matrix} \sigma_{x} \\ \sigma_{y} \end{matrix} \\ \begin{matrix} \sigma_{z} \\ \tau_{yz} \end{matrix} \\ \begin{matrix} \tau_{xz} \\ \tau_{xy} \end{matrix} \end{matrix} \right\}$ **(S1)**

There were 6 parameters, $E_{s}, E_{n}, G_{ns}, \nu_{s}, \nu_{sn}, \nu_{ns}$, among which only 5 were independent because of the symmetric condition, i.e.,

$\frac{\nu_{sn}}{E_{s}}=\frac{\nu_{ns}}{E_{n}}$ **(S2)**

The Young’s modulus $E_{n}$ along the z direction, can be obtained by using the rule of mixtures, as

$E_{n}=\frac{\rho_{GN}}{\rho_{c}}E_{c}$ **(S3)**

In the case of plane strain stretch test, a strain $\varepsilon_{y}$ was prescribed along the y direction and the two faces parallel to z-plane were constrained from moving along z direction. As such, we got $\sigma_{x}=\tau_{yz}=\tau_{xz}=\tau_{xy}=0, \varepsilon_{z}=\gamma_{yz}=\gamma_{xz}=\gamma_{xy}=0$. The $\sigma_{y}, \sigma_{z}, \varepsilon_{x}$ were extracted from the FEA calculation. From **Equation S1**, a set of equations were able to be extracted:

$-\frac{\nu_{s}}{E_{s}}\sigma_{y}-\frac{\nu_{ns}}{E_{n}}\sigma_{z}=\varepsilon_{x}$ **(S4)**

$\frac{1}{E_{s}}\sigma_{y}-\frac{\nu_{ns}}{E_{n}}\sigma_{z}=\varepsilon_{y}$ **(S5)**

$-\frac{\nu_{sn}}{E_{s}}\sigma_{y}+\frac{1}{E_{n}}\sigma_{z}=0$ **(S6)**

Since $E_{n}$ was calculated using the **Equation S3**, there were three independent variables in the **Equation S4** to **S6**. By solving these equations $\nu_{\mathrm{ns}}, E_{s},$ and $\nu_{s}$ can be determined. By combining the **Equation S2** and **S6**, the following can be obtained

$\nu_{ns}=\frac{\sigma_{z}}{\sigma_{y}}$ **(S7)**

Then from **Equation S5**

$E_{s}=\frac{E_{n}\sigma_{y}}{E_{n}\varepsilon_{y}+\nu_{ns}\sigma_{z}}$ **(S8)**

Again, from **Equation S2**

$\nu_{sn}=\frac{E_{s}}{E_{n}}\nu_{ns}$ **(S9)**

Then, by combining **Equation S2** and **S4**

$\nu_{s}=-\frac{E_{s}\varepsilon_{x}+\nu_{sn}\sigma_{z}}{\sigma_{y}}$ **(S10)**

A pure shear test in FEA simulation was used to calculate $G_{ns}$. A pure shear strain $\gamma_{xy}$ was applied between the two faces parallel to z-plane and the corresponding shear stress $\tau_{xy}$ was extracted. This leads to the shear modulus of

$G_{ns}=\frac{\tau_{xz}}{\gamma_{xz}}$ **(S11)**

The Young’s modulus of UGCM $E_{GN}$ can be calculated by a self-consistent method^5-7^. For example, following the procedures given in the literature^6^, two implicit equations need to be solved, i.e.,

$\left\{ \begin{aligned} 4\left( 18\mu+b_{1} \right)x^{2}-2\left[ 24\mu^{2}+3\left( b_{1}-2b_{2} \right)\mu-b_{3} \right]x-\left( 8b_{2}\mu+3b_{3} \right)\mu=0 \\ \left( x^{2}+b_{4}x+b_{5} \right)\left[ 2\left( 42\mu+b_{1} \right)x-\left( 3b_{1}-10b_{2} \right)\mu-36\mu^{2} \right]-4\mu\left( 6x+b_{2} \right)\left( x+\mu\right)\left( 2x+b_{4} \right)=0 \end{aligned} \right.$ **(S12)**

where $x$ was expressed as

$x=\frac{\mu\left( 9k+8\mu\right)}{6\left( k+2\mu\right)}$ (**S13)**

the coefficients were

$\left\{ \begin{aligned} b_{1}=2\left( c_{11}+c_{12}+2c_{13} \right)+c_{33} \\ b_{2}=c_{11}+c_{12}+2c_{33}-4c_{13} \\ b_{3}=3\left( \left( c_{11}+c_{12} \right)c_{33}-2c_{13}^{2} \right) \\ b_{4}=c_{44}+\frac{c_{11}-c_{12}}{2} \\ b_{5}=\frac{c_{44}\left( c_{11}-c_{12} \right)}{2} \end{aligned} \right.$ **(S14)**

$\mu$ and $k$ were the unknown shear modulus and bulk modulus of RPC. $c_{ij}$ was the element of RSC stiffness matrix, i.e., the inverse of compliance matrix shown in **Equation S1**, which can be given in terms of the previously obtained elastic parameters of RSC. The Newton-Raphson method was employed to numerically solve **Equation S12**, with initial value in terms of Hill’s approximation^8^.

1. The parameters of PDMS

The density^9^ of PDMS $\rho_{p}$ is about $1$g/cm^3^. According to reference^10^, the Poisson’s ratio of PDMS should be 0.5 but was reduced to 0.48 following the recommendation of ABAQUS user manual in order to ease the volumetric locking effect. The viscoelasticity of the PDMS can be depicted by the relaxation (e.g., shear) modulus in terms of the Prony series,

$G\left( t \right)=G_{0}R\left( t \right)$ **(S15)**

where

$R\left( t \right)=1-\sum_{k=1}^{n} g_{k}\left( 1-e^{-\frac{t}{\tau_{k}}} \right)$ **(S16)**

is the relaxation law (or the decay law). The value of each parameter in **Equation S15** is listed in **Table** **S2**. $G_{0}=G\left( 0 \right)$ is the instantaneous shear modulus and was converted into the instantaneous Young’s modulus $E_{p}=1.365MPa$ for the convenience of inputting the modulus of PDMS into ABAQUS.

***FEA models for stretchability analysis of isolated UGCM and GP-laminate***

When being compared with the isolated UGCM, GP-laminate is considered to be special at least in two aspects. The one is the presence of PDMS, which likely facilitates the load distribution within UGCM, and thus reduces the stress concentration^11^. The other is the transverse pre-compression of UGCM during assembly process, which may offer extra stretchability for the hybrid structure.

To investigate the stretchability of isolated UGCM, we modelled UGCM as a 2D Voronoi polygon model for simplicity (Fig. S1). Only one domain of UGCMS was simulated here for two reasons: 1) the direct and comprehensive modeling of the complex 3D polycrystal-like structure in FEA was too complex to handle; 2) the Voronoi polygon model was able to capture the main structure features of the UGCM networks and describe the typical deformation modes of foam-like structures, i.e., bending and stretching.

The UGCM was measured at 2.4 x 0.2 x 0.04 mm^3^ and the two PDMS layers were measured at 2.4 x 0.08 x 0.04 mm^3^. The global seeds control was set at 0.004 mm. Particularly, 0.005 mm was assigned to seeds on edges along y direction of the PDMS. This enabled the 67070 structured rectangular elements (S4R) in UGCM as well as 96000 brick elements (C3D8R) in each piece of PDMS.

To simulate the experiments, a 2-step analysis was adopted for investigating the stretchability of the isolated UGCM. The first step involves a pre-compression (40% strain) enabled at the two ends of the model by employing 4 rigid surfaces to simulate the clamping procedure before stretching. The length of rigid surface along x-direction was set equal to the thickness of UGCM along y-direction. During the second step, while fixing the y-position of the rigid surfaces, a 10% tensile strain along x-direction was subjected to the UGCM by pulling the rigid surfaces as well as the edges of UGCM that contact with the rigid surfaces away. For the GP-laminate model, a 10% strain along x-direction and -5% strain along y direction were applied, simultaneously, to mimic the Poisson’s effect of PDMS groove upon the resulting structural stretchability.

***FEA model for UGCM-PDMS interfacial bonding analysis***

Considering the complex structure of UGCM, we treated the UGCM here as a continuum and built it in to a sandwiched structure with an outer PDMS layer (Fig. S2). The UGCM layer was placed into the groove formed by the PDMS layer, which was further extended by 1.2 mm to act as a “handle”. The extending loads along x-direction are applied on the exterior y-surfaces of the handle. Only a quarter of the specimen was studied in this case owing to the two symmetrical planes of the GP-laminate system, i.e., x = 0 and z = 0. The sizes of the UGCM layer and PDMS groove shown in Fig. S25 was 12 x 0.4 x 4 mm^3^ and 12 x 0.8 x 6 mm^3^, respectively.

The model was meshed with brick elements (C3D8R), 93120 elements in total. The global seed size was 0.1mm. Particularly, the seed size of PDMS layers along y-direction was 0.05mm, so that at least four layers of elements were meshed even in the thinnest part. The seeds on every edge were carefully controlled and the elements were strictly constrained to seeds, so that nodes on all the interfaces exactly matched with their counterparts, and thus merged together if both sides of the interface were PDMS.

The interface between the UGCM and PDMS was assumed to be perfectly bonded, which was modelled by tying each node on the interface to its precisely matching counterpart.

The displacement load with smooth step amplitude was applied on the top and bottom faces of the “handle” along x direction. The displacement of one node on the interface between PDMS groove and the handle was monitored in order to calculate the loading strain. The total nodal force components along loading direction (herein x) on the two interfaces parallel to y-plane were also extracted to compute the mean interface shear force.

The experimental density and Young’s modulus of UGCM are taken from the reference^4^. According to these experiments, an empirical relation between the density $\rho_{\mathrm{GN}}({mg/cm}^{3}$) and Young’s modulus $E_{\mathrm{GN}}\left( \mathrm{MPa} \right)$ of UGCM could be obtained, i.e., $E_{\mathrm{GN}}=k\rho_{\mathrm{GN}}^{2}$, with $k=5.3211\times{10}^{-4}MPa/\left( {mg/cm}^{3} \right)^{2}$.

The UGCM layer was considered as a composite of cell walls and void. To ascribe its appropriate properties, the net volume of cell walls in our Voronoi polygon model was calculated by ABAQUS. The 95% compression during assembly only caused the volume decrease of voids, with little change to the volume of the cell walls and as a result, the volume ratio of cell walls increased. By the rule of mixtures, the average density could be expressed by the density and volume ratio of cell walls, and the average Young’s modulus by the Young’s modulus and volume ratio of cell walls. The parameters of UGCM layers obtained following the above-mentioned procedures are listed in **Table S3**.

***Analysis of the UGCM density effect upon the real contacting areas between the UGCM and PDMS***

In this section, we will use some geometry analysis to show that for the GP-laminate structure, the real contact area A_con_ (for a given interfacing area) between the UGCM and the PDMS layer should be proportional to the density ρ of the UGCM. In light of the honeycomb-like structure of the UGCM, we used a model structure shown in Fig. S26 to do the analysis. Two key parameters were defined for the honeycomb structure, including the lateral dimension of the hexagon (L) and the thickness of the solid wall (t). From the Fig. S26a, the ratio of the contacting area over the nominal interfacing area can be derived as

$\frac{A_{con}}{A int}= \frac{t}{L}$ **(S17)**

The porosity of the foam can be calculated from the Fig. S26b as

$f=\frac{\left( \frac{L-t}{2} \right)^{2}}{{\frac{L}{2}}^{2}}=\left( 1-\frac{t}{L} \right)^{2}=1-\frac{2t}{L}+({\frac{t}{L})}^{2} \sim1-\frac{2t}{L}$ **(S18)**

Given the density of graphene wall is ρ_0_, the ρ of the UGCM can be derived as

$={}_{0}*\left( 1-f \right)={}_{0}*(\frac{2t}{L})$ **(S19)**

Given the geometries of the UGCMs with different density are the same, the A_int_ can be treated as a constant, therefore, the A_con_ between the graphene foam and PDMS should be proportional to the ρ. It is reasonable to expect a higher contact area will lead to a stronger bonding. Therefore, the interface strength should be proportional to the ρ.


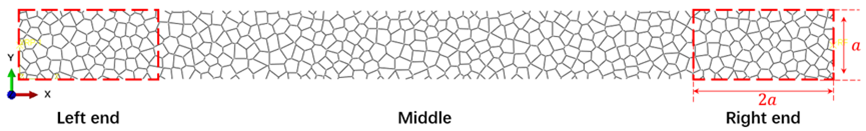
**Fig. S1. A FEA demonstration of UGCM, modelled by a 2D Voronoi polygon model structure, for the stress concentration calculation.** The UGCM was divided into 3 parts and allocated into two groups - the group end and the group middle. The rectangular regions within 2 times of the length of UGCM along y-direction away from both ends were grouped as the end points, while the rest was labelled as the middle. The stress concentration factor (SCF) was defined as the ratio of max principal stresses of the end and middle group. More modelling details are illustrated in Materials and Method.

The SCF under the applied strain of isolated UGCM and UGCM assembled in the GP-laminate are shown in Fig. 1a. The average SCF of the isolated UGCM was around 10 with a minimum value of 5.5 and maximum value reached 20. The stress in the isolated UGCM was found to be highly concentrated near the two ends where clamps were applied. This is consistent with conventional understanding from solid mechanics that stress concentration often takes place at the area where geometric or boundary condition suddenly changes. This high SCF value of the isolated UGCM could explain its low stretchability observed during the experimental tensile tests (in our experiments, the isolated UGCM always broke at the clamped regions under uniaxial stretching) (Fig. S3).

By contrast, GP-laminates demonstrated a more uniform stress distribution along the UGCM network. This is mainly due to the outer PDMS layer able to delocalise these concentrated stresses^11,12^ and provide uniform and effective protection to the UGCM. The SCF of GP-laminate was around 1 with a maximum value of 1.2. Therefore, the UGCM within the GP-laminate can possibility be stretched to higher strain limit upon the same exterior loading than the UGCM alone.

**
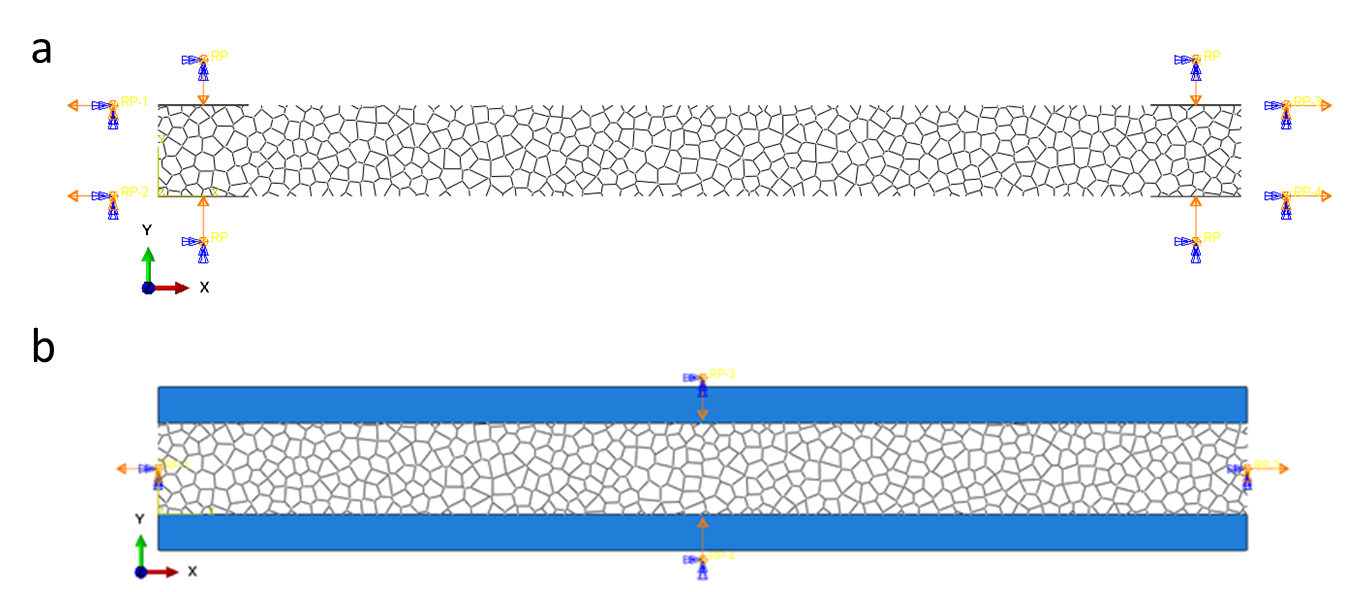
Fig. S2. The adopted FEA model structures of UGCM and GP-laminate for stretchability investigation.** **(a)** A 2D Voronoi polygon model represented the UGCM network. The UGCM was measured as 2.4 x 0.2 x 0.04 mm^3^. **(b)** The GP-laminate model, representing the UGCM, was sandwiched in between two PDMS layers (blue layers). The UGCM and PDMS layers were measured as 2.4 x 0.2 x 0.04 mm^3^ and 2.4 x 0.08 x 0.04 mm^3^, respectively.


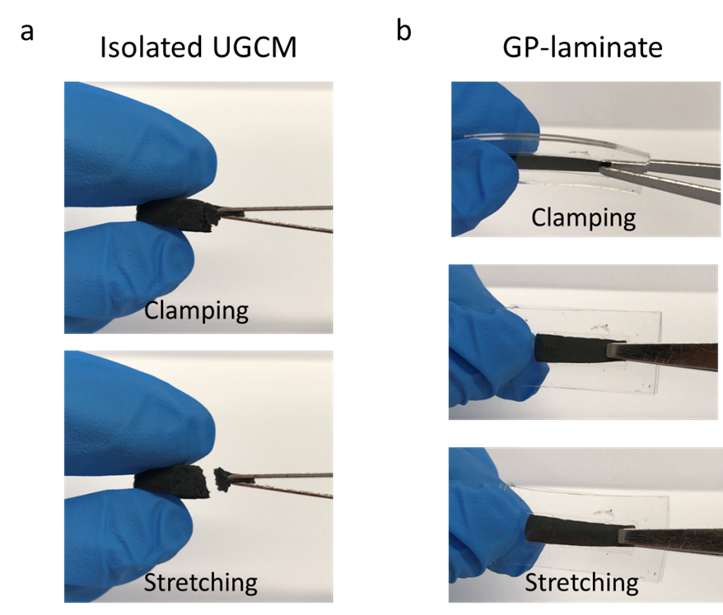


**Fig. S3. Photos of clamping and stretching UGCMs with a tweezer with and without PDMS protective layers.** **(a)** Photos of clamping (top) and stretching (bottom) an isolated UGCM with a tweezer. These results indicate that the clamping or direct use of UGCM lead to severe localised cracking at the clamping area. **(b)** Photo illustration of GP-laminate structure with enhanced clamping- and deforming-tolerance that the hybrid can be easily clamped and stretched by a tweezer without structural ruptures. The densities of the UGCMs are around 1.0 mg/cm^3^.


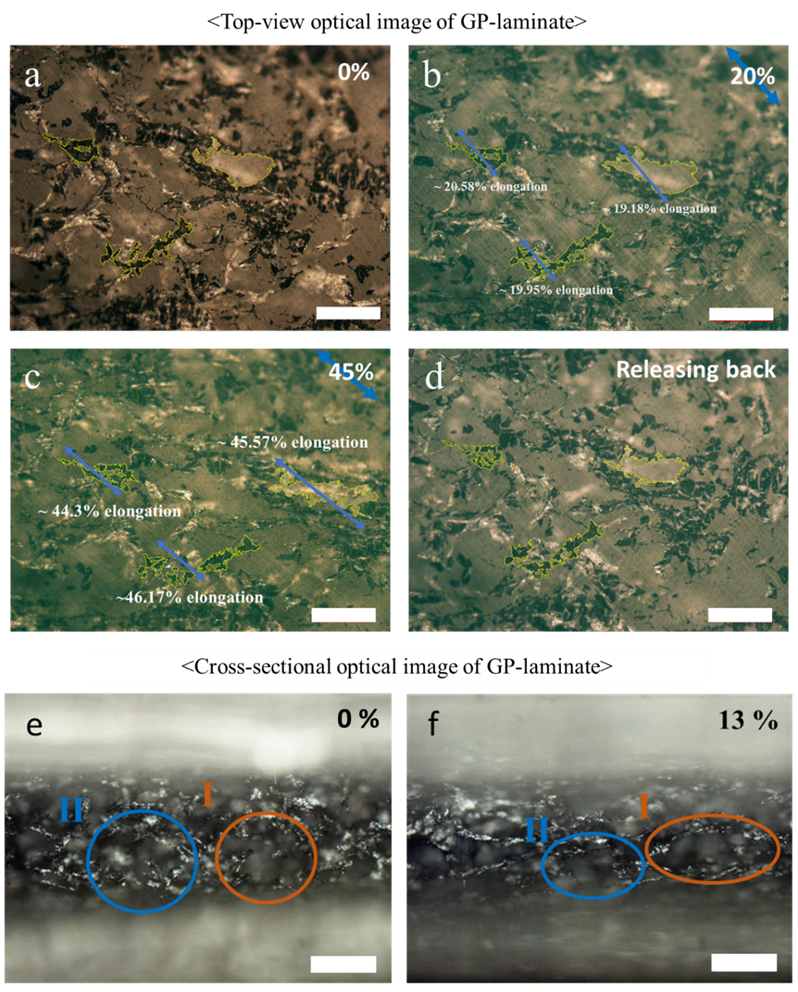


**Fig. S4. Optical characterisation of the internal microstructure of GP-laminate under stretching.** Top-view **(a-d)** and cross-sectional **(e-f)** optical microscopic images of the GP-laminate under different stretching conditions. The hybrid was subjected to tensile strain from 0% up to 45% (a-c) and releasing (d) at the tenth loading/unloading cycle. The density of UGCM is 1.0 mg/cm^3^. There was very little detachment observed at the interface. (e-f) Cross-sectional optical microscopic images of the UGCM/PDMS lamellar hybrid underwent strains from e) 0% to f) 13%. Scale bar: 100 μm.

The internal microstructure morphologies of the assembled GP-laminates were characterised using a laser optical microscope (VK-9700, Keyency). A home-made clamping device was attached to the stage of the microscope with two in-built screws to apply the strain so that the microstructure can be examined *in-situ* under the deformation. The optical microscopic images were analysed by the ‘Image J’ software to determine length changes, shape changes and the aerial changes of the images.

It can be seen that, when subjected to a uniaxial tensile strain, the assembled UGCMs within the hybrid structure were uniformly deformed along the strain direction at both the interfacial areas (Figs S4a to S4d) and the middle sections (Figs S4e and S4f). To characterise the structural change of the hybrid during cycling deformation, the optical microscopic characterisation was performed after ten stretching/release cycles. Figs S4a to S4d illustrate the sequential images captured from the top view of the hybrid with the strain of 0%, 15%, 45% and then releasing back. The black areas in the images are the interfacial regions of the UGCM and PDMS, while the relatively transparent areas are internal porous structures of the UGCM. Three typical areas were selected for observation of their elongation during the tensile deformations. When the hybrid was subjected to a uniaxial strain, both the UGCM-PDMS interfacing areas and the internal porous structures of UGCM were found to be uniformly stretched. When the external strain was released, the UGCM-PMDS interfacing area was able to restore its original state, with negligible detachment at the interface. These connecting points of UGCM and PDMS could possibly serve as the pivots that allow effective stress transfer from the PDMS to UGCM and thus where these stresses can be transferred within the UGCM network due to its structural coherence resulting from the strong intersheet interaction between graphene sheets.

Moreover, such uniform stretching condition can uniformly distribute the tensile loading to the UGCM, leading to the uniform crack generation inside the UGCM and further enhance the stretchability of the GP-laminate. Figs S4e and S4f show the cross-sectional optical microscopic images of the fabricated hybrid, the hybrid was sequentially deformed from 0 to 13% (maximum strain of the home-made device) and then released. The two highlighted areas are typically suggesting the uniform conformable deformation of UGCM in the GP-laminate when subjected to the tensile deformation. A schematic illustration of the crack generations and reconnections in UGCM is shown in Fig. S5.

It can be seen that during tensile deformation in Figs S4e and S4f, the interlayer distance between the two PDMS layers was reduced when stretched up to 13%. This could be the reason for the occurrence of a slight deviation of the hysteresis curve shown in Fig. 1c, because of a coupling effect of the crack generation along the stretching direction and the squeezing of UGCM under stretching.

**
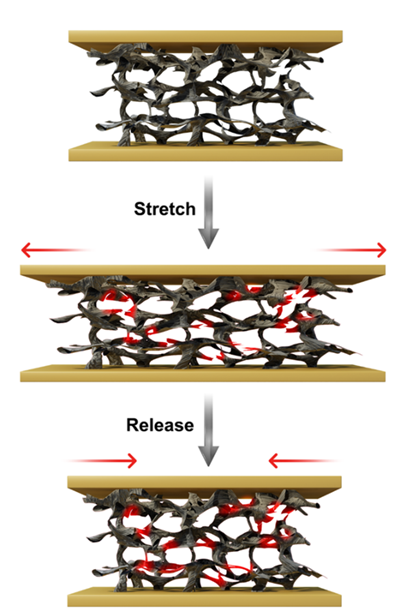
**

**Fig. S5. Schematic of a possible crack sensing mechanism of the GP-laminate when subjected to cyclic tensile deformation.** The reversible microcracks generation of UGCM networks (highlighted by red) with strain deformation, can enable the strain-induced resistance changes of the hybrid owing to the high conductivity of UGCM. Ideally, when a cyclic external tensile strain is applied, the GP-laminate is expected to provide an increase in its electrical resistance in response to the applied strains due to the formation of cracks (disconnections and disruption of connectivity of the graphene structure). When the applied strains are released, the deformed UGCM is able to recover along with the PDMS, leading to the reconnection of the scissored structure and thus restoration of resistivity.


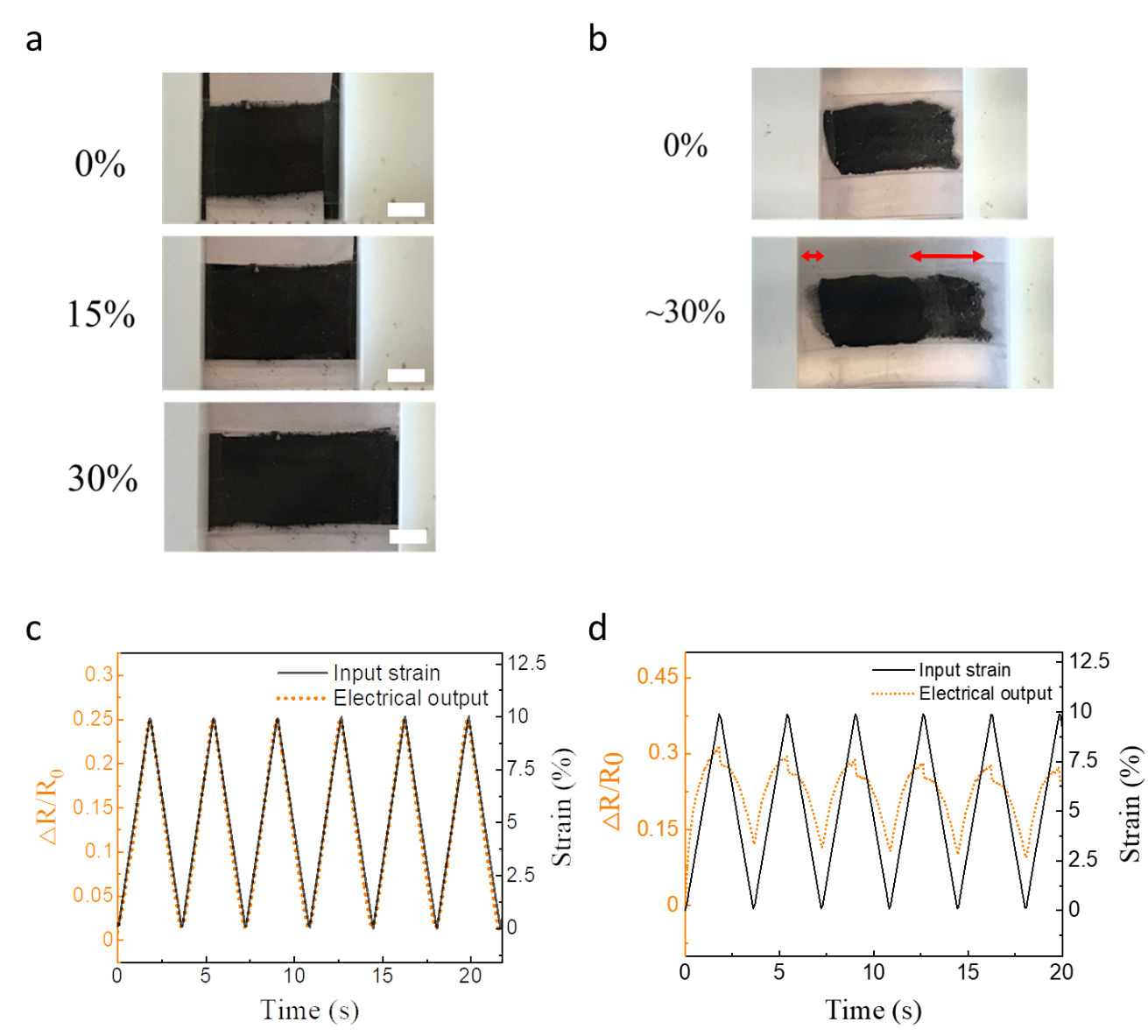


**Fig. S6.** **Mechanical and electromechanical tensile performance of GP-laminates assembled with different densities of UGCM under tensile tests.** Photos of GP-laminates fabricated from UGCMs with densities of **(a)** 1.0 mg/cm^3^ and **(b)** 5.0 mg/cm^3^ when being stretched from 0 to 30% strain. The slip areas between the UGCM and PDMS are marked with a red arrow showing a light grey colour indicative of only a very thin layer of graphene remaining on those areas of the PDMS layer. The relative change in resistance of GP-laminates assembled with different densities of UGCM of **(c)** 1.0 mg/cm^3^ and **(d)** 5.0 mg/cm^3^ in response to the cyclic tensile deformations. The externally applied strains were set at 10%, with a strain rate of deformation 5 mm/s.

It can be seen that when deformed, no obvious cut-through cracks or fractures along with the UGCM networks was found in the GP-laminate with the density of 1.0 mg/cm^3^, while significant slippage between the higher density UGCM (5.0 mg/cm^3^) and PDMS occurred. This resulted in the stretching of PDMS layers but nonuniform and limited length extension of the UGCM (5.0 mg/cm^3^). These phenomena have been further proven by the electromechanical tests (Figs S6c and S6d). Hybrids with lower density UGCM (1.0 mg/cm^3^) showed reversible and cyclic changed strain-dependent resistance changes. However, hybrids with higher density of UGCM showed a non-linear and irreversible response between the relative resistance changes and the externally applied strains, which is likely caused by slippage at the UGCM-PDMS interface. Note that other GP-laminates assembled from UGCM with densities of 2.5 mg/cm^3^ and 3.0 mg/cm^3^ have also been fabricated and tested, and likewise showed similar slippage phenomena.


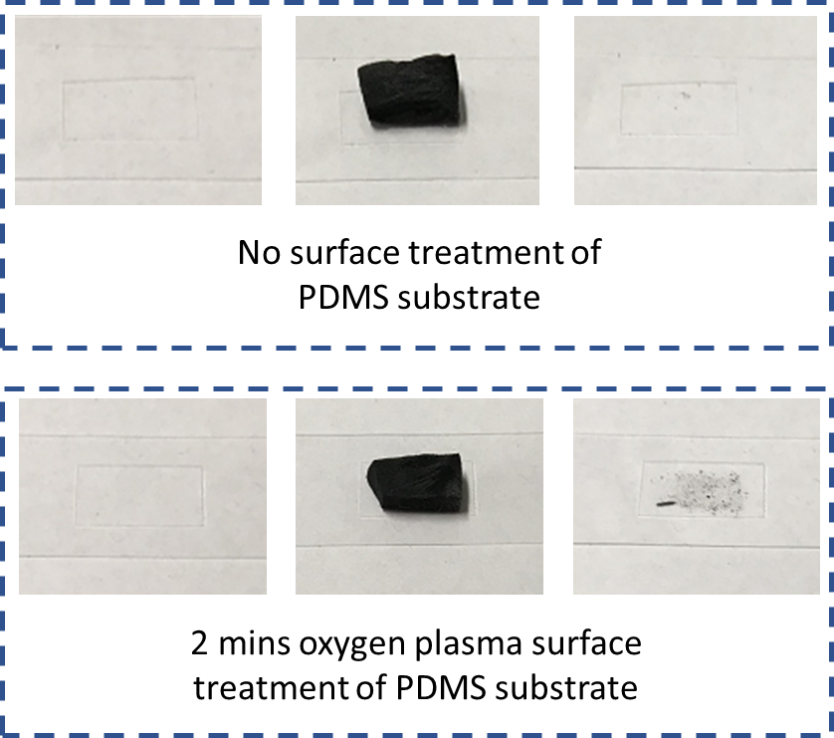


**Fig. S7. Images illustrating the interfacial interactions between UGCMs and PDMS layers with and without oxygen plasma surface treatment of PDMS layers.** After peeling of UGCM, a thin layer of graphene remaining on the oxygen plasma treated PDMS, while a clean surface was found in the pristine PDMS. This suggests enhanced interfacial bonding introduced by the treatment. The enhanced interfacial bonding strength is possibly due to two reasons. The first involves the replacement of the relatively stable methyl groups (- CH_3_) with the silanol groups (- OH) upon the repeated units of – o – Si (CH_3_)_2_ – of the PDMS layers when being treated with the oxygen plasma**^13^**. Note that the graphene sheets used in the UGCM networks were rGO, rather than pristine graphene. It is thus possible that the more reactive silanol group can bond with the remaining functional groups on the rGO sheets of the UGCMs. In addition, the surface bonding changes of the PDMS layers may also be attributed to the nano-structuring of the PDMS surfaces. Additionally, after oxygen plasma-treated treament, rough PDMS surfaces can be observed**^13,14^.** This may help to create physical interlocking between rGO and PDMS layers**^15^.** Noteworthy, we found that varying the oxygen plasma treatment duration of the PDMS layers did not show any further significant change in the stretchability of GP-laminates, especially for higher density UGCMs.


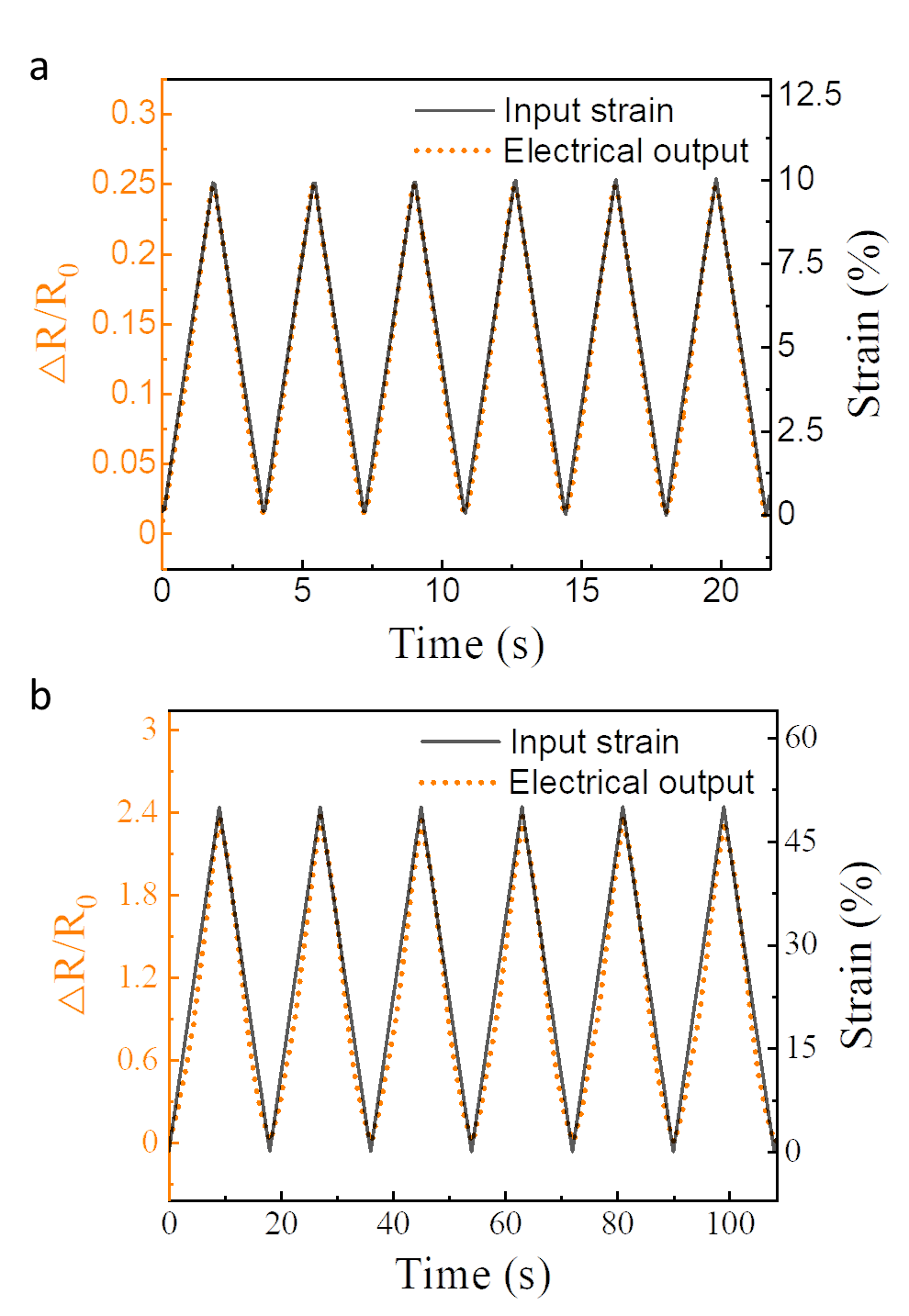


**Fig. S8. Electromechanical response of GP-laminate to varied strains.** Relative resistance changes of GP-laminate in response to periodic tensile loading cycles under strains (black curve) of **(a)** 10% and **(b)** 50%. The strain rates were set at 5 mm s^-1^.


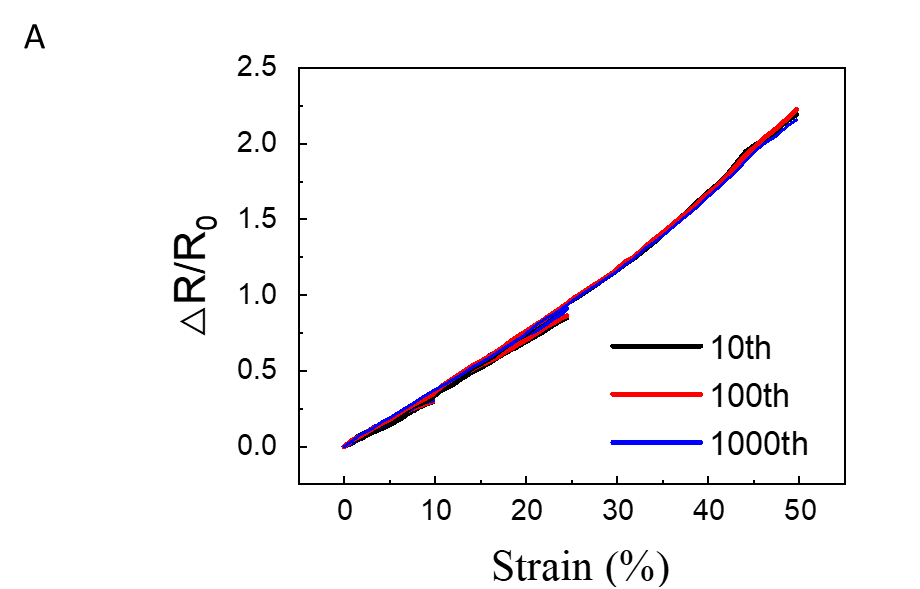


**Fig. S9. Durability characterisation of GP-laminates.** Relative changes in resistance of the GP-laminate versus applied strains for 10 cycles (black), 100 cycles (red) and 1000 cycles (blue) at different strain levels varied from 10 % to 50%. The tests suggest that the GP-laminate provides nearly unchanged strain-response for over 1000 cycles at strain up to 50%, indicating that the sensors can fully recover after repetitive loading/releasing cycles.


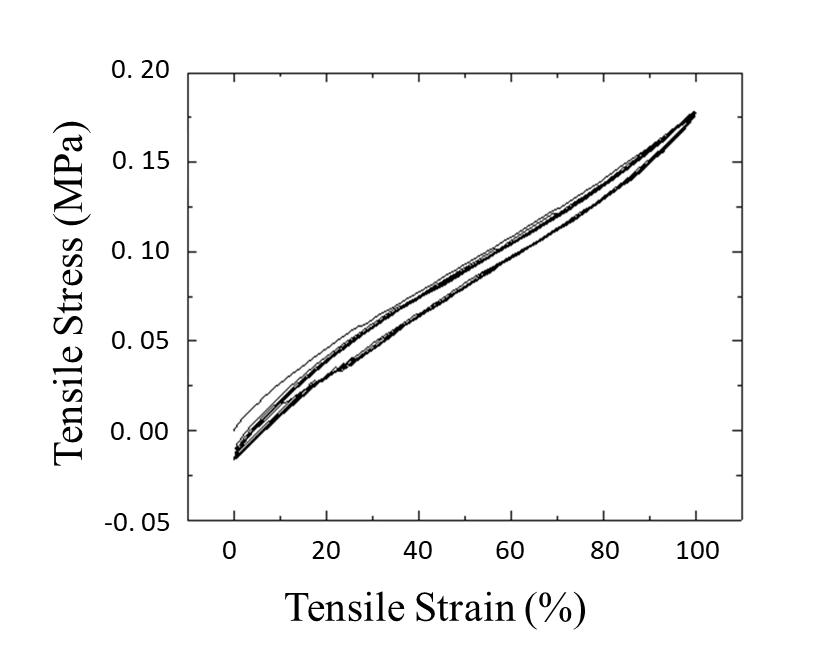


**Fig. S10. Typical stress-strain curve of GP-laminate.** The GP-laminate possesses the excellent stretchability up to 100% elongations with low modulus of around 250 kPa, similar values to that determined for the human epidermis (130 kPa to 300 kPa)**^16,17^**.


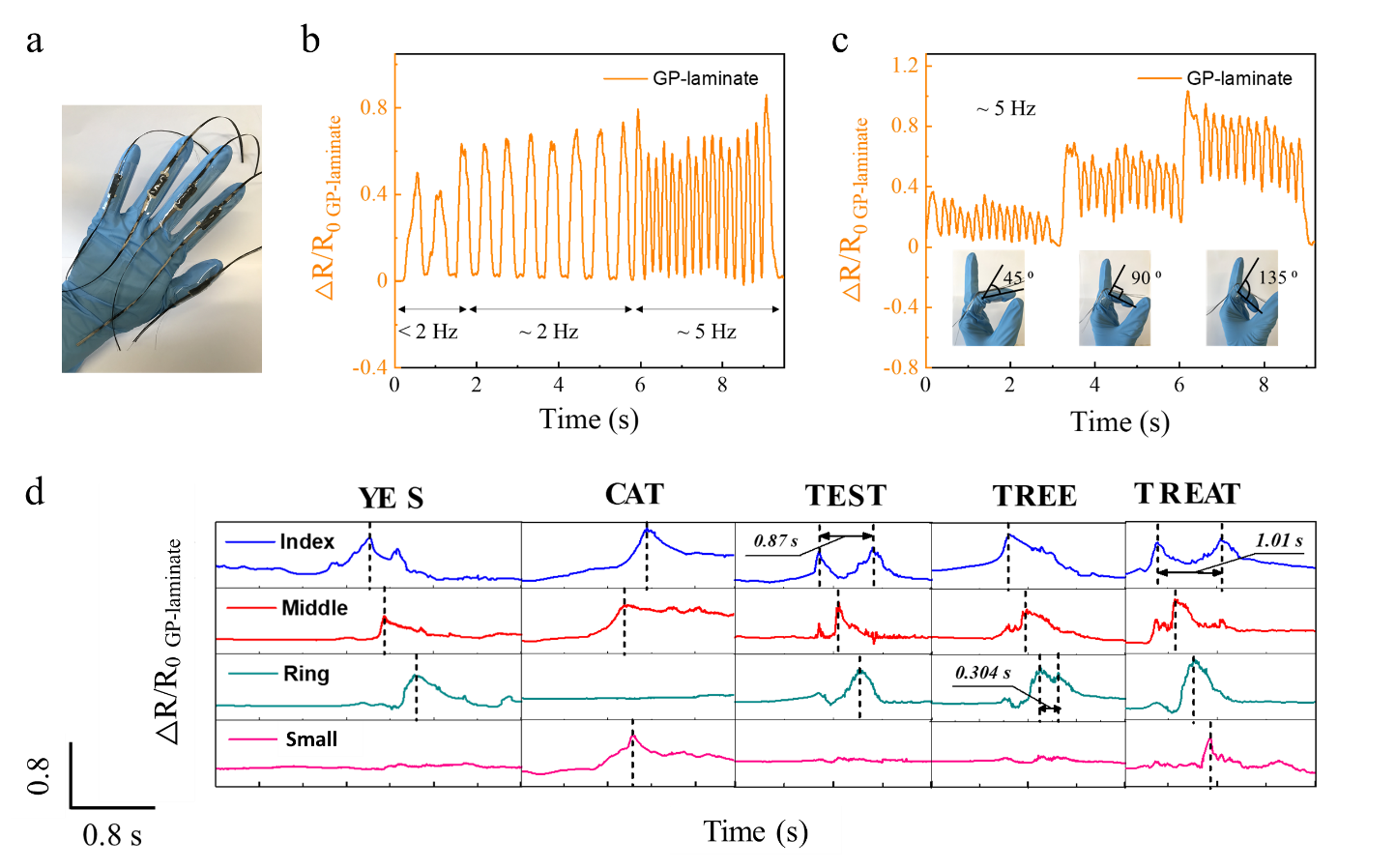


**Fig. S11. Using GP-laminate for resistive sensing of finger movements.** **(a)** Photograph of a data glove mounted with five GP-laminates. The relative change in resistance of the GP-laminate on the index finger for detecting finger bending motions in response to **(b)** varied frequencies and **(c)** different bending angles at a given frequency (~ 5 Hz). **(d)** Relative changes in resistance of GP-laminates versus time for detecting fast left-hand side typing behaviour of the subject.

The GP-laminate was used to monitor the joint activities, one of the most studied bodily physical activities in the literature^18-21^. To assemble the GP-laminates-based data glove, five single GP-laminates were used to be mounted onto the five fingers of a lab glove. A commercial flexible adhesive bandage was used to fix the GP-laminates to the glove to allow the GP-laminate to conform to every contortion or deformation of the gloves during finger movements. Distinct from the literature where the frequencies tested were mostly lower than 1 Hz, this test was performed in a higher frequency range up to 5 Hz. A strain sensor-based data glove was developed to monitor the fast finger movements (Fig. S11a). The subject wore the data glove to perform the finger motions for different bending angles and speeds.

The subject wore the data glove on their left hands, and firstly performed different cyclic bendings of the left-hand index finger with gradually increased cyclic bending speed from lower frequencies (< 2 Hz) to higher frequencies (> 5 Hz). It was found that the bending and straightening of the fingers were picked up by the GP-laminate by showing the upwards and downwards curves of its relative change in resistance (Fig. S11b). These resistance changes followed quite well with the different bending speeds of the fingers. Moreover, it was found that the GP-laminate can demonstrate different bending angles of the index finger by illustrating different amplitudes of their changes in resistance. As shown in Fig. S12c, at a relatively high bending speed of the finger with the cyclic bending frequency at nearly 5 Hz, the maximum relative resistance changes of the strain sensors increased significantly from 0.4 to 1, with an increase of the finger’s bending angles from 45 º to 135 º. These results indicate that the GP-laminate can conform to every contortion or deformation of the gloves during finger movements, also indicating the potential of using GP-laminates for precisely monitoring and quantifying high-speed (up to 5 Hz) finger movements.

The capability of precisely monitoring finger motions is important for a range of applications. For example, in recent years, realising hand and finger gesture recognitions for enabling a range of emerging technologies, such as tracking professionals’ finger movements**^22,23^**, or soft robotics development**^18^** via wearable electronics have attracted great attention, but still remains unsolved problems for extracting accurate and instantaneous hand/finger information from both fast and slow speeds, with wearable, efficient, low cost and easy readout systems. We then used a GP-laminate-based data glove to monitor the typing behaviour of the subject. It was found that information related to the typing speed, the moving range of each finger and the coupling effects between each finger can be read out easily from the corresponding electrical signals. For example, when typing the word ‘TREE’, the left ring finger provided very fast motions but with limited motion range during the double hit of the letter ‘E’. And when typing the word ‘YES’, large motion range of the left index finger was required to reach the letter ‘Y’ located in the middle part of the keyboard. Moreover, the coupling effect between fingers can also be seen, especially when typing words like ‘TEST’, ‘TREE’ and ‘TREAT’. For example, when typing the first capital ‘T’ by the index finger, the response from the middle and ring fingers can also be observed. These results suggest that the complex finger motions can be fully captured by using the GP-laminates at broadband strain and speed ranges, which could provide more critical information for a range of novel applications, especially related to the athletic and professional performance analysis and human-computer interaction.


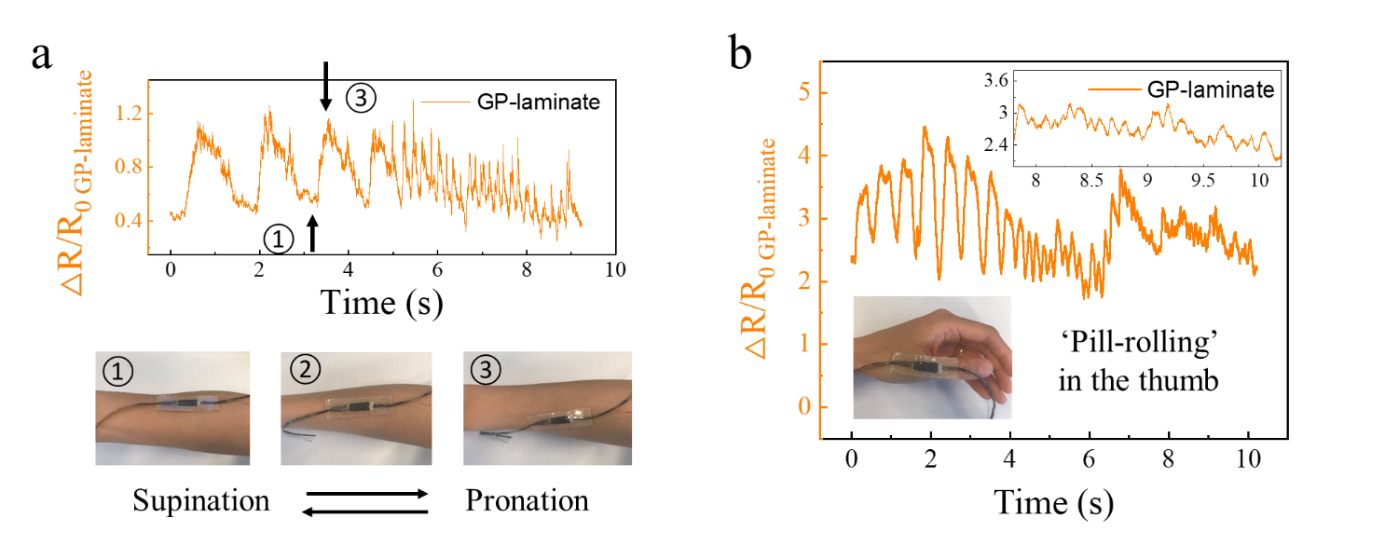


**Fig. S12.** **Using GP-laminate for monitoring mimicked movement disorders.** **(a)** and **(b)** Parkinsonian tremor detections manifesting as **(a)** Pronation or supination in the forearm and **(b)** ‘pill-rolling’ in the thumb. The insets in **(a)** show the arm position changed from supination to pronation with corresponding signals highlighted in the resistance changes of the sensor. The bottom left inset in **(b)** demonstrates the ‘pill-rolling’ disorder detection by using the strain sensor in the thumb. The top right inset in **(b)** shows the zoom-in the output signal of the strain sensor within the last 2 s.

Tremor, including the rest tremor and postural tremor, is the most common movement disorder^24^. For example, approximately 70% of suffers of Parkinson’s disease (PD) present with a tremor symptom. Tremors are generally be grouped into rest tremor and postural tremor. The rest tremor frequency is typically located in 3 – 6 Hz, and the postural tremors can reach up to 12 Hz**^24,25^.** It was found that by attaching the GP-laminate onto different body parts, the mimicked movement disorders were able to be detected by this hybrid. As can be seen in Fig. S12a, when placing the strain sensor on the forearm of the subject, the transitions of the forearm from the supination status to the pronation status were detected by the GP-laminate. Such resistance changes were also able to be detected by the strain sensor on the forearm with frequencies higher than 5 Hz. Fig. S12b illustrates the mimicked ‘pill-rolling’ behaviour of the thumb. By simply attaching the GP-laminate to the thumb joint via an adhesive bandage, the strain sensor provided distinctive electrical signal readouts with varied frequencies greater than 7 Hz. These results indicate the potential of using GP-laminate for movement disorder detection, offering an alternative low-cost, flexible and simple methodology for movement disorder assessments.


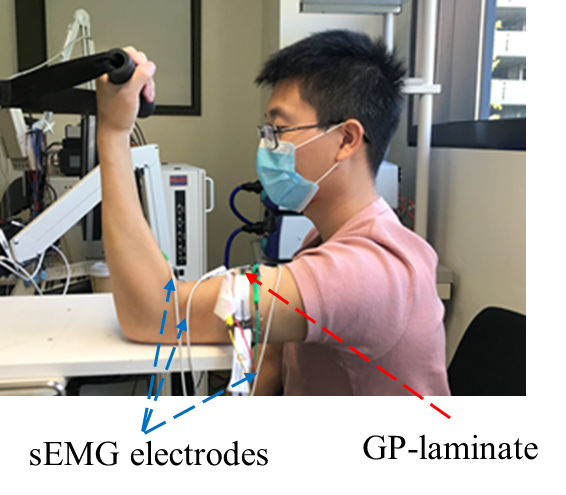


**Fig. S13. A photo of attaching GP-laminate and sEMG on to the bicep muscle group for skeletal muscle contraction detection.** The GP-laminate was placed on the belly area of the bicep muscle group. Two non-elastic bands (orange bands) were connected to the two ends of the GP-laminate. The whole device was tightened around the arm.

**
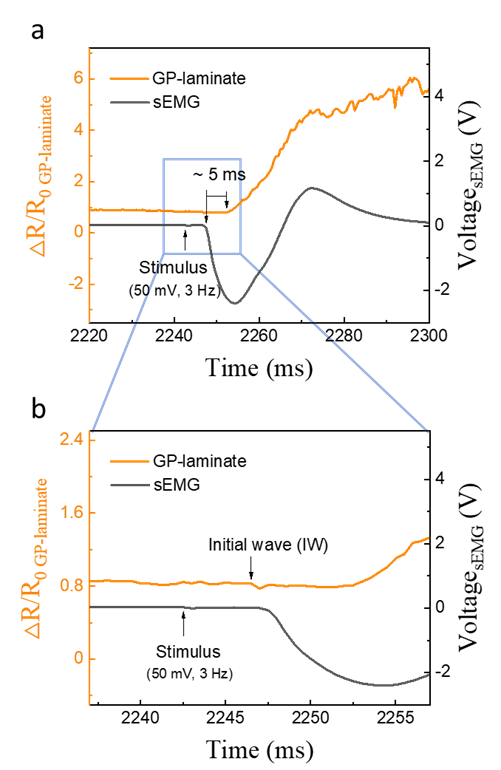
**

**Fig. S14. The typical signal recordings from the GP-laminate and a sEMG for monitoring the evoked bicep muscle contraction.** **(a)** The resistance change recording (orange curve) and the muscle action potential recording (black curve) from the target biceps muscle group following a direct stimulation of the target peripheral nerve at Erb point. The supramaximal stimulation intensity was set at 50 mA and 0.2 ms duration with a stimulation frequency set at 3 Hz. The latency between the two signals was calculated from the two highlighed onset points (pointed by the arrows). The onset of stimulation was also illustrated by an arrow prior to the onset of the electrical response detected by the sEMG. This is due to the conduction along the terminal nerve from the point of stimulation to the synapse and then the synaptic transmission onto the target muscle^26^. Such stimulation-sEMG delay observed during our experiment is around 4.5 ms, in good agreement to the literature^26,27^. **(b)** The signal recordings from **(a)** at a higher amplification. The GP-laminate shows a negative ‘initial wave (IW)’ prior to the demonstration of the onset of the strong recording resistance changes of the strain sensor.

As emphasised previously, the EMG only reflects the electrical rather than the mechanical events of the muscle contraction. During the electro-mechanical coupling effect that induce the muscle contraction, EMG only represents the propagation of motor unit action potentials along muscle fibres, which serving as the initiator of the muscle fibre shortening. Such time span between the electrical events and the actual muscle contraction is defined as the electromechanical latency (EML)^28,29^. Details of the measured EML using the GP-laminates are illustrated in Fig. S15.

Fig. S14b indicates the IW detected by the GP-laminate. This IW phenomenon has so far been observed and confirmed in MMG, and was generally described as a small preceded wave change of the recording signal showing an oppsite polarity to the main strong recording peak^28^. This IW indicates a minute relaxation (termed as latency relaxation, LR) of the stimulated muscle group prior to the beginning of muscle fiber tension rise^28,30,31^. It has also been reported that such LR is possibly due to the lengthening of the sarcomere in the latent period of the skeleton mucle group after the stimulation^31^. The detection of such subtle muscle fibre movements indicates the high sensitivity of the GP-laminates for monitoring human sketetal muscle activities.

It is worth noting that, from the sensitivity perspective, the GP-laminates are not as ‘sensitive’ as previously reported extremely sensitive stain sensors**^15,32-34^**. As can be seen in Fig. 1c, the detected gauge factor (GF), which is given by GF = (△R/R_0_)/strain, of the hybrid is no larger than 6, which is much smaller than that of most existing extremely sensitive strain sensors. It seems that, in more specific scenario, such as monitoring the subtle skeletal muscle activities, strain sensors are required to possess not only moderate GF but also high signal-to-noise ratio and soft mechanical compliance (Fig. S10) to skin.


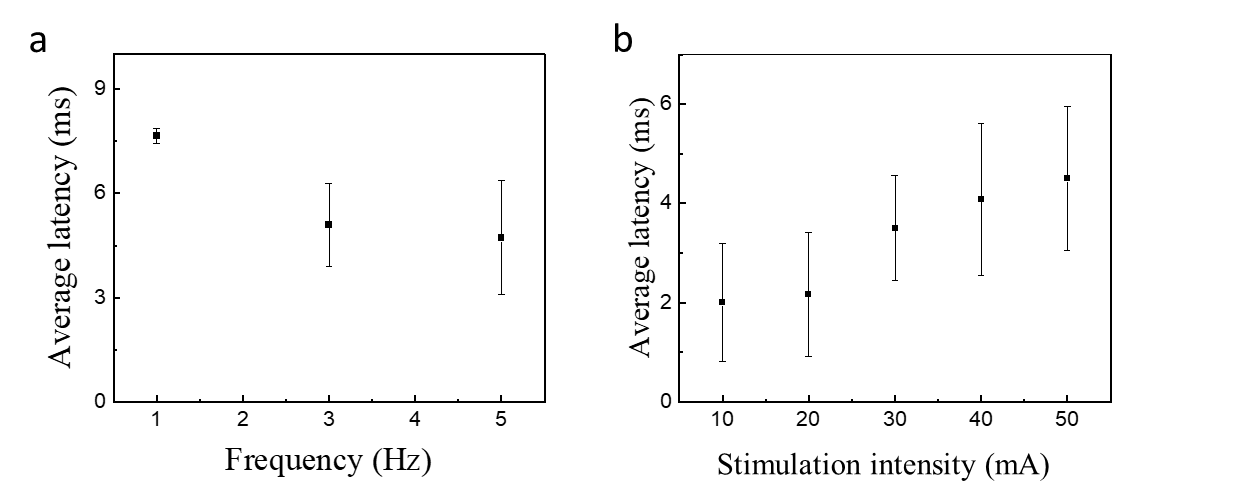


**Fig. S15. The average latencies between the two output signals of the GP-laminate and the sEMG when synchronously subjected to monitor the evoked biceps muscle contractions.** **(a)** The recorded signal average latencies (average of 6 trails) between the two sensors when monitoring the evoked bicep muscle contractions at different stimulation frequencies of 1 Hz, 3 Hz, and 5 Hz. The stimulation intensity was set at 50 mA. **(b)** The recorded average signal latencies (average of 6 trials) between the two sensors when monitoring the evoked bicep muscle contractions at different varied stimulation intensities of 10 mA, 20 mA, 30 mA, 40 mA and 50 mA. The stimulation frequency was 3 Hz.

The EML performed by the GP-laminates under different stimulation conditions have been illustrated here. Fig. S13a indicates the frequency-dependence of the EML recorded from the resistive changes of the GP-laminate during the evoked muscle contractions. It was found that the EML decreased with the increase in stimulation frequency. However, to the best of our knowledge, this phenomenon has not been reported previously by using other types of MMG-transducers and is likely contributed by muscle fibres types, including Type I (reaching peak force at a slow speed), Type IIa (reaching peak force more rapidly) and Type IIb (producing the highest amount of force within a shortest time period) ^35^, may be triggered differently by the given stimulation with different frequencies and result in different respond speed to the applied stimulus. Fig. S15b illustrates the intensity-dependence of the EML recorded from the resistive changes of the GP-laminate during the evoked muscle contractions. It is shown that the EML increased with the increase of stimulation intensity, which shows the opposite trend demonstrated in the literature measured the by a microphone^28^, which is worthy of further investigation. Overall, the measured EML from the evoked bicep muscle contraction detected by the GP-laminate varied from ~1 to 7.6 ms falls in the value range (1 to 37 ms) previously reported using other types of transducers^29^.

It is worth noting that, by using different transducers and clinical protocols, the EML results in the literature were inconsistent**^35-38^.** It has been widely accepted that the magnitude of these time spans in a given motor task may be determined by a series of factors of the device resolution, data processing, and the structural differentiation of the neuromuscular system, i.e. muscle fibre types, etc**^35-38^**. This demonstrates the need for different technologies in the near future for comprehensive detections of complex muscle activities. In this work we propose the GP-laminate as an alternative strategy that offers the combined advantages to complement current existing MMG-transducers. For example, the accelerometer has rarely been reported to pick up muscle activities within very short EML (< 6 ms) and has rarely been reported to detect the subtle LR phenomenon, probably due to its relatively large self-weight that could fall onto the muscle and offset the actual muscle vibrations**^29,39^**. On the contrary, by picking up the subtle muscle vibration through an acoustic signal may result in larger EML (> 37 ms) due to device limitations**^29^**. The GP-laminates can provide both high sensitivity and narrow EML during muscle contraction detections.

**
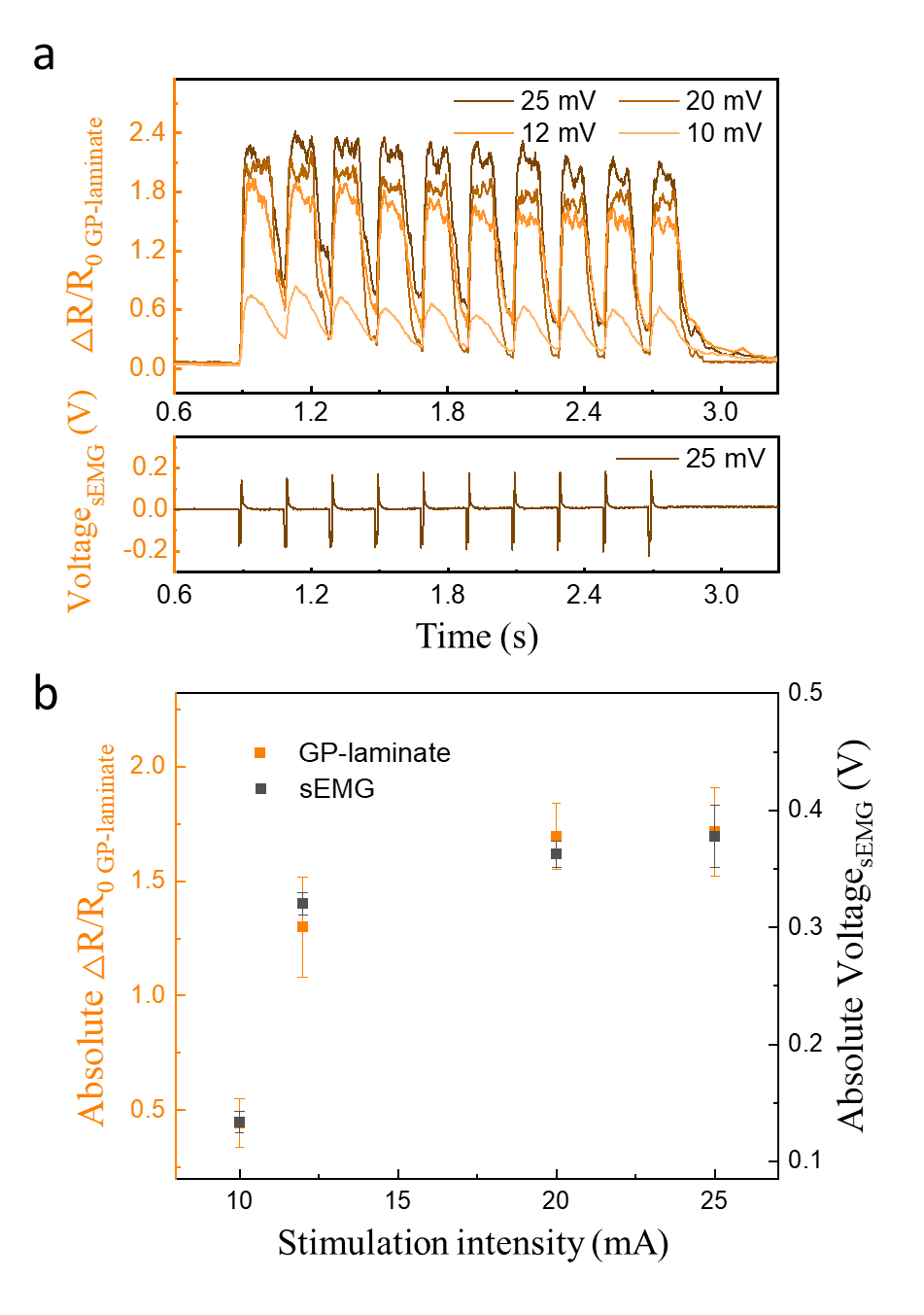
**

**Fig. S16.** **Using GP-laminate for monitoring the evoked wrist flexor muscle contractions.** **(a)** The relative change in resistance of the strain sensor for monitoring stimulated wrist flexor muscle contractions at varied stimulation intensities of 25 mA, 20 mA, 12 mA and 10 mA. The stimulation frequency used was 5 Hz. The simultaneous sEMG recording at the stimulation intensity of 25 mA is shown in the bottom as a reference. **(b)** A comparison of the absolute relative change in resistance of the GP-laminate (orange dots) and the absolute voltage change of the sEMG (black dots) in response to the monitored stimulated wrist flexor muscle contractions at different given stimulation intensities. The variation in these two signal outputs, indicated as error bars, were calculated from 9 multiple tests recorded by 3 different GP-laminates with similar gauge factors.


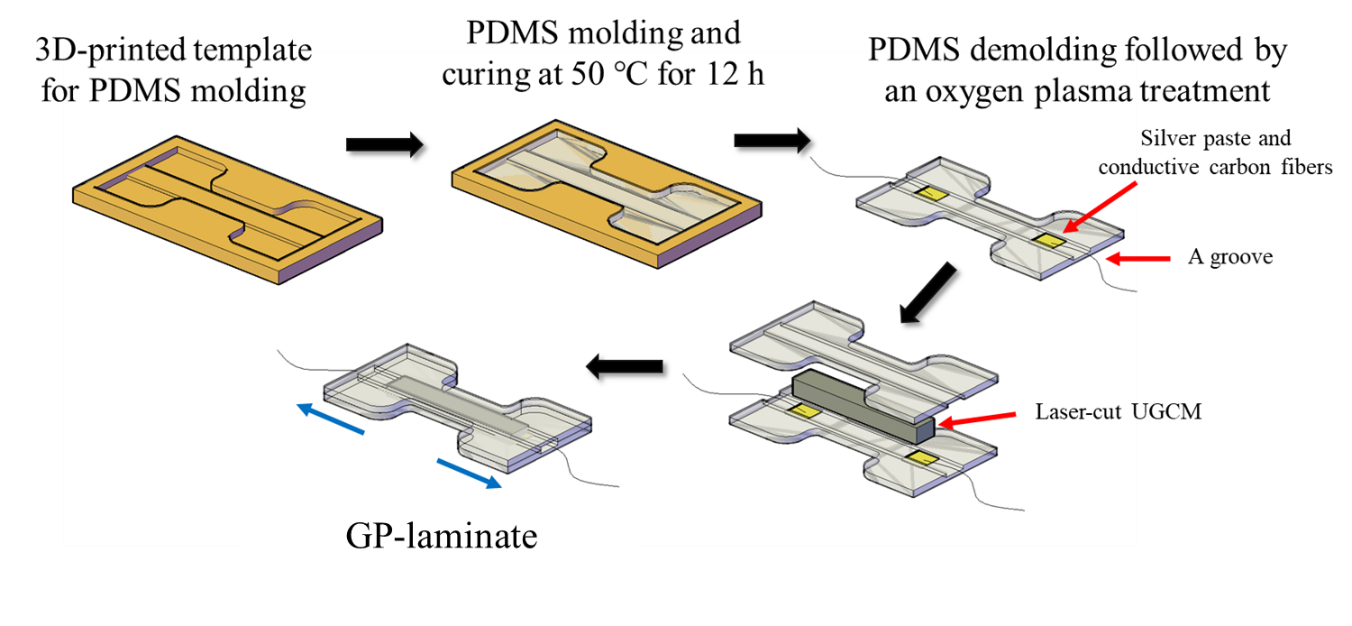
**Fig. S17. Schematic illustration of detailed fabrication process for assembling GP-laminate with in-built grooves on the PDMS layers.**

**
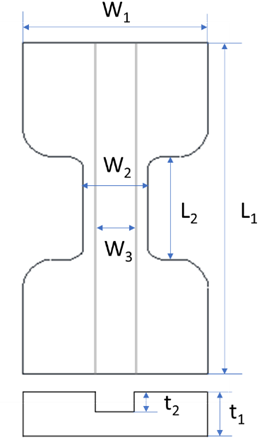
**

**Fig. S18.** **The detailed design parameters of PDMS substrates for assembling GP-laminate for mechanical tensile tests.** The width (W_1_) = 35 mm and length (L_1_) = 70 mm. The width of the dog-bone neck (W_2_) = 14 mm. For cases with in-built grooves, the width of the groove (W_3_) = 10 mm. The thickness of the PDMS (t_1_) and groove (t_2_) were chose at 400 μm and 200 μm, respectively.


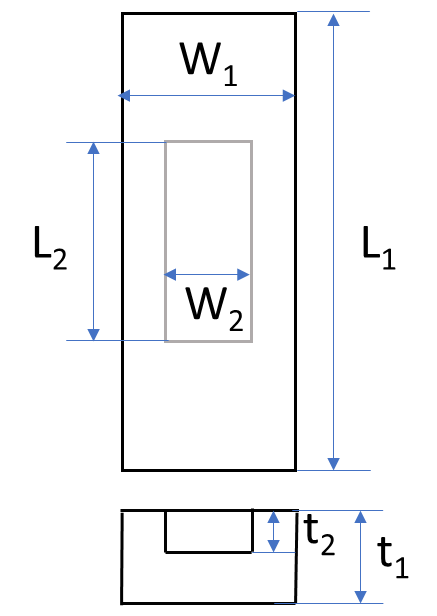


**Fig. S19. The detailed design parameters of PDMS substrates for assembling GP-laminate for skeletal muscle activities detections.** The width (W_1_) = 12 mm and length (L_1_) = 30 mm. The width of the in-built grooves (W_2_) = 10 mm. The length of the in-built grooves (L_2_) = 20 mm, which was 2 mm shorter than the laser cut UGCM to allow enough connects of the UGCM with the silver paste at its two ends. The thickness of the PDMS (t_1_) and groove (t_2_) were 400 μm and 200 μm, respectively.


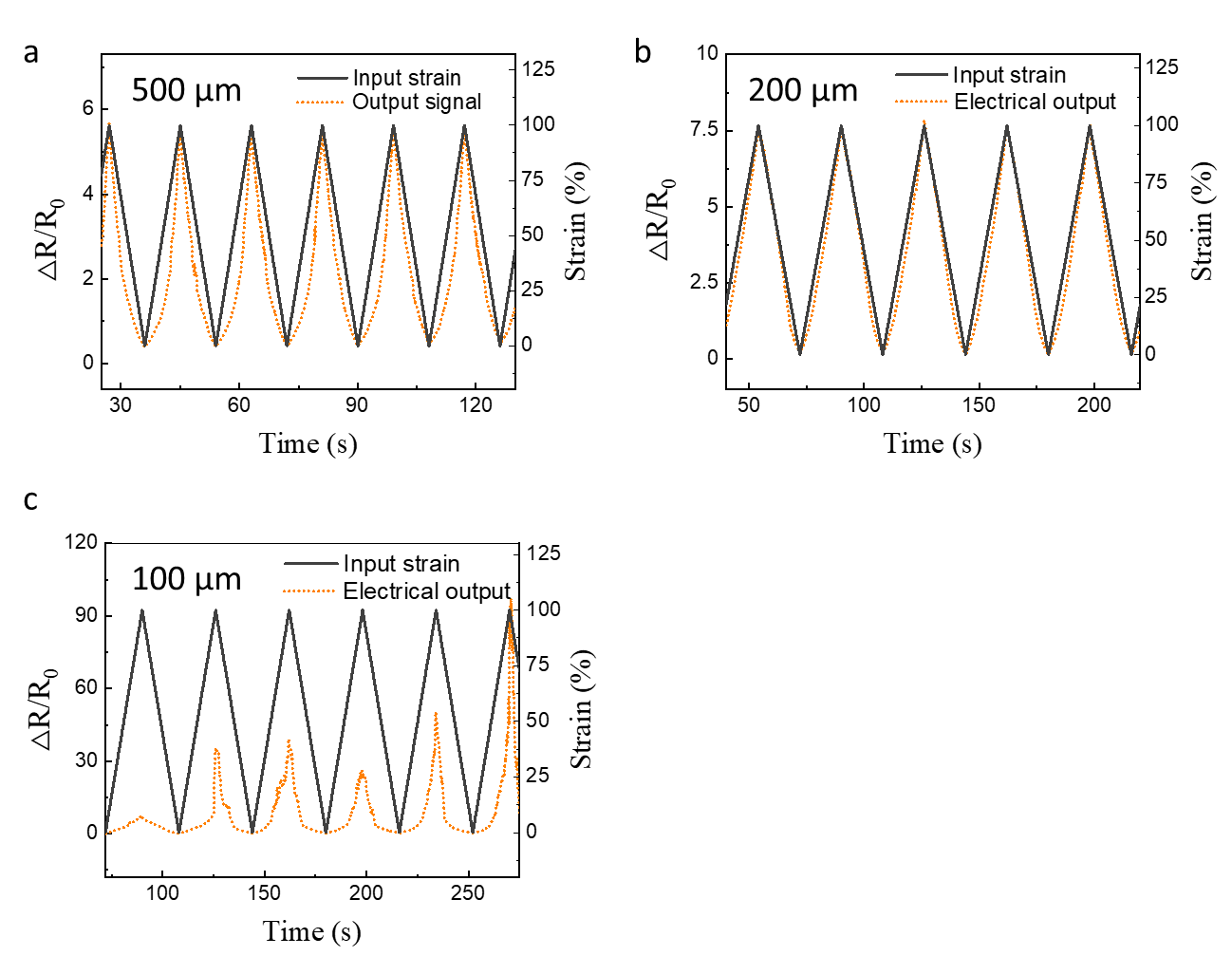


**Fig. S20. The effect of PDMS thickness.** Typical changes in resistance of GP-laminates assembled from PDMS layers with different thicknesses of **a)** 100 μm, **b)** 200 μm and **c)** 500 μm, respectively, in response to different applied tensile strains at 100%. The applied strain rate was 0.5 mm/s. The assembled UGCMs possessed a density of 1 mg/cm^3^. The interactions between UGCMs and PDMS were generated via a 2 min oxygen plasma surface treatment of PDMS layers.

We found that when the thickness of the PDMS layers was reduced from 500 μm down to 100 μm, the electrical response of the GP-laminates changed significantly. Thick PDMS layers always caused strong compression to the UGCMs and resulted in less effective resistance change of the graphene network especially under stretching due to the conformal contacts between graphene sheets. This strong compression-induced relative strain-insensitivity was further demonstrated by the relatively smaller relative change in the resistance value of the 500 μm-PDMS-layer assembled hybrid, which is around 5.2, than that (~ 7.6) of the 200 μm-PDMS-layer hybrids when being stretched with 100% strain. In contrast, GP-laminates assembled from extremely thin PDMS layers also suffered with non-linear electromechanical response. This could be attributed to the extremely low modulus of thin PDMS layers which cannot press the UGCMs uniformly within the assembling structure and cause the non-uniform cracking inside the UGCM networks. These results indicate the critical influence of the degree of the precompression of the UGCMs within the hybrid structures, on their electromechanical property. We then modified the GP-laminate by introducing an in-built groove on to the PDMS substrates to change the degree of the pre-compressions upon the UGCMs within the hybrid structures. The details design parameters are shown in Figs S18 and S19.


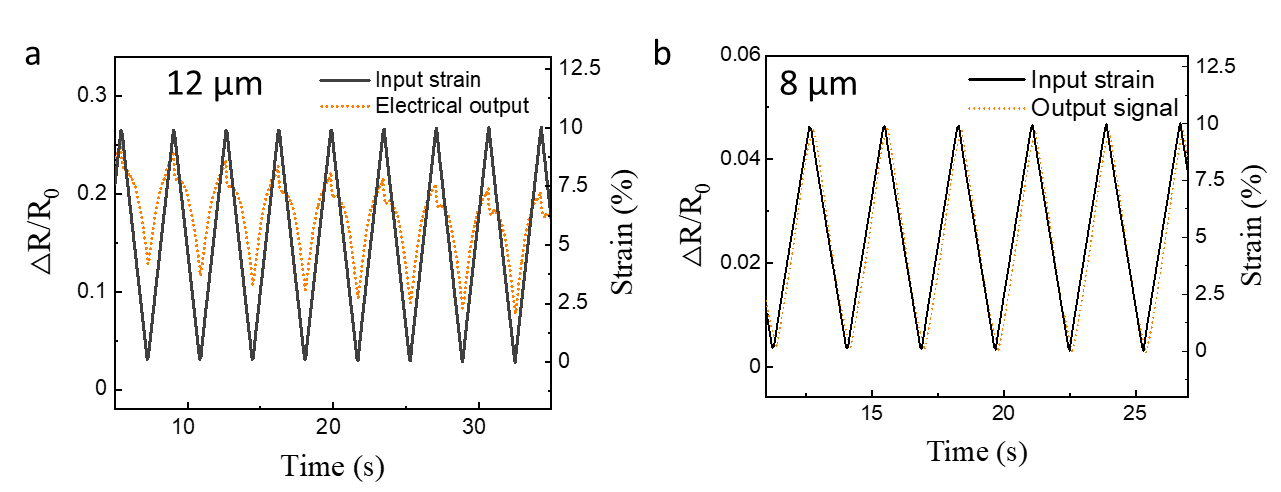
**Fig. S21. The effect of UGCMs thickness**. Typical changes in resistance of GP-laminates assembled from UGCMs with different thicknesses of **a)** 12 μm and **b)** 8 μm, respectively, in response to different applied tensile strains at 10%. The applied strain rate was 0.5 mm/s. The assembled UGCMs possessed a density of 1 mg/cm^3^. The interactions between UGCMs and PDMS were generated via a 2 min oxygen plasma surface treatment of PDMS layers.

The pre-compression of the UGCMs within the hybrid structure can also be tuned by adding different amounts and thicknesses of UGCMs in to the PDMS grooves. Fig. S21 demonstrates how would the thickness of the assembled UGCMs cause effect onto the electromechanical property of the hybrids. It can be seen in Fig. S21a, that hybrids with a very thick layer (*i.e.*, 12 μm) of UGCMs, whilst able to provide cyclic resistance changes, can lead to a non-linear relationship between resistance and the applied strains, and an electrical signal decay can be observed. In contrast, hybrids assembled from 8 μm-thick-UGCMs show a highly linear response between the input and output signals. This issue is likely caused by the less effective stress transfer from the PDMS layer to the UGCM networks, since when the UGCM is very thick, limited surface interactions are not conducive to provide efficient shear stresses to enable the uniform stretching of the enclosed UGCM. In addition, the reduction of resistances during the cyclic loading/unloading cycles is also likely due to the sliding occurred at the interfaces between UGCM and PDMS. The UGCMs with thinner layers of 6 mm were also produced but the thin UGCMs always resulted in poor stretchability and poor signal responses. As reported previously, this could mainly be due to the lower tolerance to the defects/cracks of the thinner UGA-based composite materials.

**
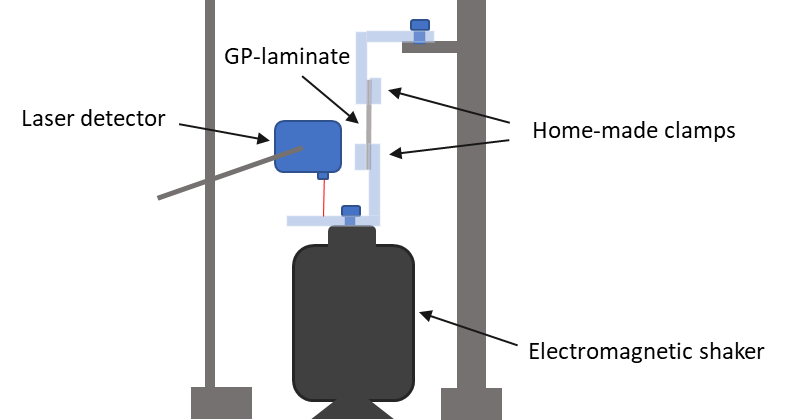
**

**Fig. S22. The tensile test setup for GP-laminate for the high-frequency dynamic deformation tests.** The GP-laminate was clamped to an electromagnetic shaker which can provide accurate, high-frequency vibrations under high frequency by a home-made clamp with two in-built screws at one end. The other end of the GP-laminate was fixed to an immobile holder with another similar designed home-made clamp. The applied strain deformations onto the strain sensor were detected by a laser detector which detected the up-down movements of the shaker. Note that the detection limit of the laser detector is around 25 Hz, therefore the applied strain was not illustrated in Fig. 2d for the 180 Hz cyclic test.

**
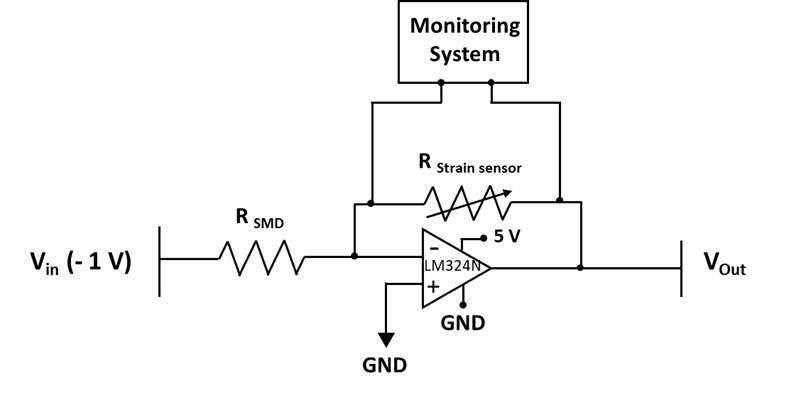
**

**Fig. S23. The readout circuit based on an op-amp (Texas Instruments, LM324N) to detect the electromechanical response of the GP-laminate under high-frequency deformations.**

***
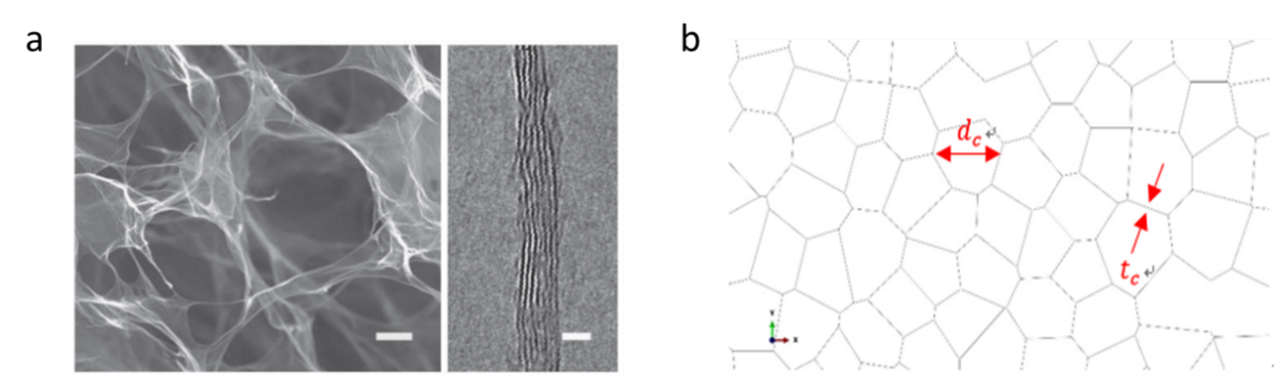
***

**Fig. S24. UGCM structure applied for FEA modelling. (a)** SEM image^4^ (left) of UGCM with a density of 1.1 mg/cm^3^. The scale bar represents 10$\mu m$. TEM image (right) of the cell wall thickness**^4^**. The scale bar represents 2 nm. **(b)** A Voronoi polygon model used to model a typical domain in UGCM in FEA simulations.


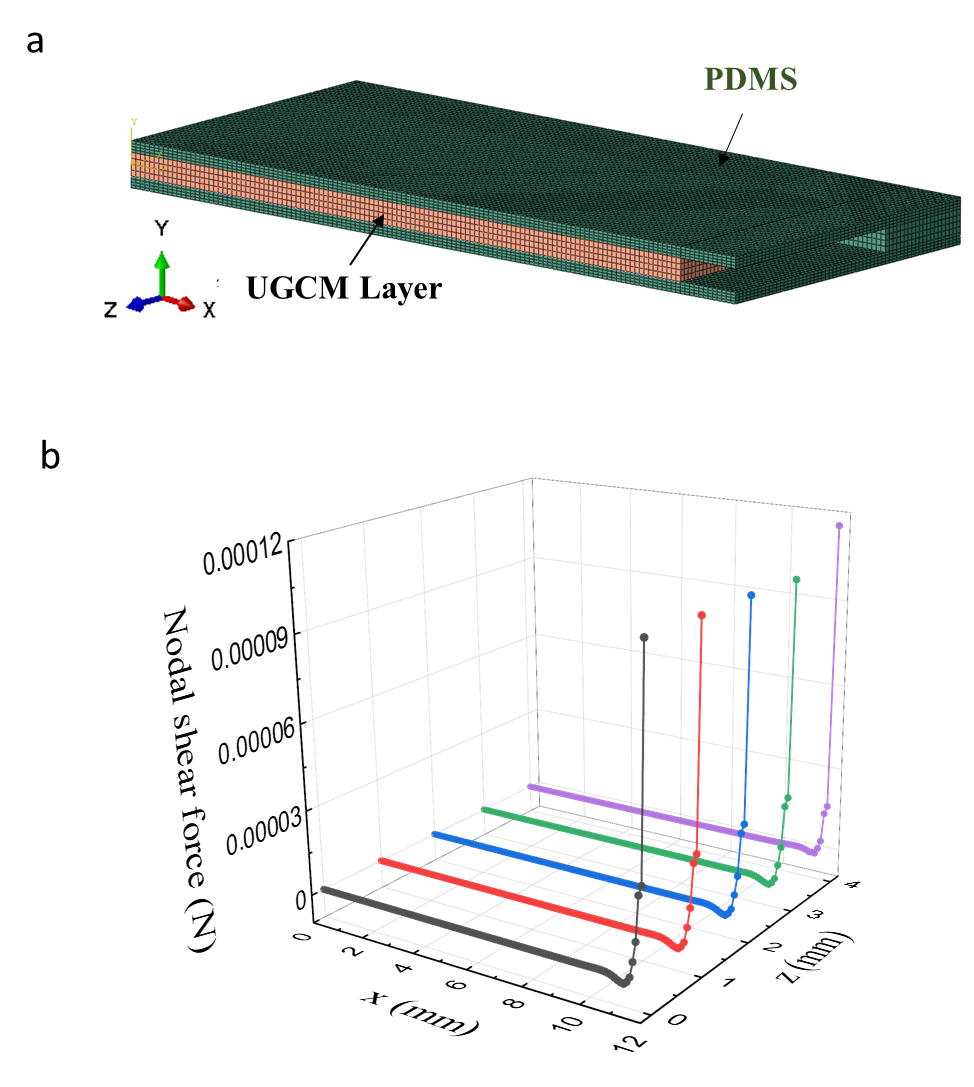


**Fig. S25. The FEA model adopted for UGCM-PDMS interfacial force analysis.** Only a quarter of the specimen was employed owing to its two symmetrical planes, i.e., x = 0 and z = 0. The sizes of the UGCM layer and PDMS groove models shown in the present Fig. were 12 x 0.4 x 4 mm^3^ and 12 x 0.8 x 6 mm^3^, respectively.

**
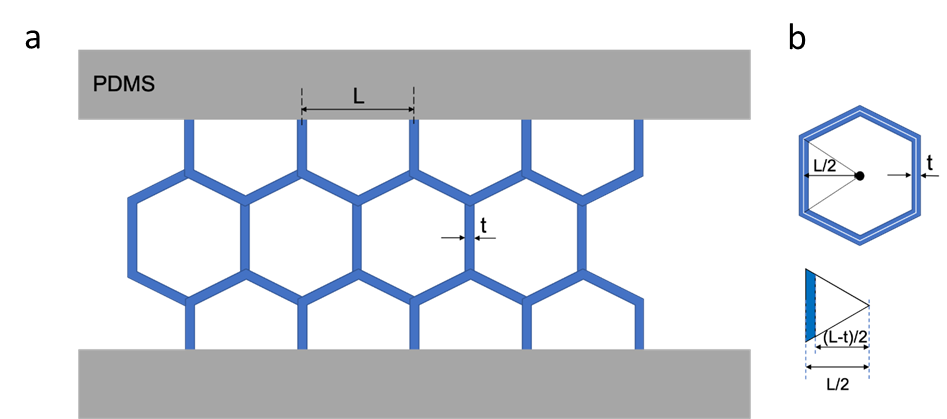
**

**Fig. S26. Schematic illustration of the sandwich structure of the GP-laminate.** **(a)** A schematic structure of the GP-laminate assembled by sandwiching a honeycomb UGCM network in between two PDMS layers. The lateral dimension of the repeat hexagon unit of the honeycomb and the thickness of the solid network cell wall are defined as L and t, respectively. **(b)** The repeat hexagon unit of the honeycomb structure for assembling the UGCM networks for the porosity analysis.

Table S1. Estimations of cell wall Young’s modulus of UGCM with different densities.

| Density of UGCM  $\rho_{GN}$  (mg/cm^3^) | Young’s modulus of cell walls  $E_{c}$  (GPa) | Thickness of cell walls  $t_{c}$  (nm) | Young’s modulus of UGCM  $E_{GN}$  (kPa) | |
| --- | --- | --- | --- | --- |
|  |  |  | Empirical calculation ^a^ | FEA ^b^ |
| 0.5 | 32 | 1.82 | 0.13 | 0.12 |
| 1.1 | 21 | 4.00 | 0.64 | 0.54 |
| 5.1 | 14 | 18.60 | 13.8 | 13.8 |
| 7.0 | 12 | 25.46 | 26.1 | 21.4 |

^a^ Empirical calculation: To reduce the random error in experimental measurement, the Young’s modulus of the UGCM recorded in the literature^4^ were fitted to $E_{\mathrm{GN}}=k\rho_{\mathrm{GN}}^{2}$. The empirical calculated $E_{GN} (MPa)$ is calculated by substituting $\rho_{GN} {(mg/cm}^{3})$into this empirical relationship. Note the $k=5.3211\times{10}^{-4}MPa/\left( mg/\mathrm{cm}^{3} \right)^{2}$.

^b^ FEA: To model the multi-domain and multi-orientation structure of UGCM, we modelled one domain with cross-section as 2D Voronoi polygons, and then obtain the FEA Young’s modulus of UGCM by self-consistent method.

**Table S2. The adopted parameters in Prony series of PDMS**^10^.

| Parameters | Values |
| --- | --- |
| $G_{0}$ | 0.455MPa |
| $g_{1}$ | 0.08 |
| $\tau_{1}$ | 0.165s |
| $g_{2}$ | 0.03 |
| $\tau_{2}$ | 5s |

**Table S3. Parameters of UGCM layers composed by UGCMs with different densities.**

| Density of original UGCM  (mg/cm^3^) | Density of resultant UGCM layer  (t/mm^3^) | Young’s modulus of resultant UGCM layer  (MPa) |
| --- | --- | --- |
| 1.0 | $2.2\times{10}^{-11}$ | 0.01288 |
| 2.5 | $5.0\times{10}^{-11}$ | 0.06650 |
| 3.0 | $6.0\times{10}^{-11}$ | 0.09576 |
| 5.0 | $1.02\times{10}^{-10}$ | 0.27680 |

**SI References**

1 Coskun, M. B. *et al.* Ultrasensitive Strain Sensor Produced by Direct Patterning of Liquid Crystals of Graphene Oxide on a Flexible Substrate. *ACS Appl Mater Interfaces* **8**, 22501-22505, doi:10.1021/acsami.6b06290 (2016).

2 Tarata, M. T. Mechanomyography versus electromyography, in monitoring the muscular fatigue. *Biomed Eng Online* **2**, 3, doi:10.1186/1475-925x-2-3 (2003).

3 Esposito, D. *et al.* A Piezoresistive Sensor to Measure Muscle Contraction and Mechanomyography. *Sensors (Basel)* **18**, 2553, doi:10.3390/s18082553 (2018).

4 Qiu, L., Liu, J. Z., Chang, S. L., Wu, Y. & Li, D. Biomimetic superelastic graphene-based cellular monoliths. *Nature communications* **3**, 1241, doi:10.1038/ncomms2251 (2012).

5 Kroner, E. Berechnung der elastischen Konstanten des Vielkristalls aus den Konstanten des Einkristalls. *Zeitschrift fur Physik* **151**, 504-518, doi:10.1007/bf01337948 (1958).

6 Min, L. & Zheng, L. Y elastic constants of polycrystalline materials with hexagonal system structure. *Acta Physica Sinica* **58**, 8511, doi:10.7498/aps.58.8511 (2009).

7 Yaguchi, M. & Busso, E. P. On the accuracy of self-consistent elasticity formulations for directionally solidified polycrystal aggregates. *International Journal of Solids and Structures* **42**, 1073-1089, doi:10.1016/j.ijsolstr.2004.07.009 (2005).

8 Hill, R. The elastic behaviour of a crystalline aggregate. *Proc. phys. soc. A* **65**, 349, doi:10.1088/0508-3443 (1952).

9 Delamarche, E., Schmid, H., Michel, B. & Biebuyck, H. Stability of molded polydimethylsiloxane microstructures. *Advanced materials* **9**, 741-746, doi:10.1002/adma.19970090914 (1997).

10 Lin, I. K. *et al.* Viscoelastic Characterization and Modeling of Polymer Transducers for Biological Applications. *Journal of Microelectromechanical Systems* **18**, 1087-1099, doi:10.1109/jmems.2009.2029166 (2009).

11 Lu, N., Wang, X., Suo, Z. & Vlassak, J. Metal films on polymer substrates stretched beyond 50%. *Applied Physics Letters* **91**, 221909, doi:10.1063/1.2817234 (2007).

12 Li, T. & Suo, Z. Ductility of thin metal films on polymer substrates modulated by interfacial adhesion. *International Journal of Solids and Structures* **44**, 1696-1705, doi:10.1016/j.ijsolstr.2006.07.022 (2007).

13 Bodas, D. & Khan-Malek, C. Hydrophilization and hydrophobic recovery of PDMS by oxygen plasma and chemical treatment—An SEM investigation. *Sensors and Actuators B: Chemical* **123**, 368-373 (2007).

14 Tan, S. H., Nguyen, N. T., Chua, Y. C. & Kang, T. G. Oxygen plasma treatment for reducing hydrophobicity of a sealed polydimethylsiloxane microchannel. *Biomicrofluidics* **4**, 32204, doi:10.1063/1.3466882 (2010).

15 Amjadi, M., Turan, M., Clementson, C. P. & Sitti, M. Parallel Microcracks-based Ultrasensitive and Highly Stretchable Strain Sensors. *ACS Appl Mater Interfaces* **8**, 5618-5626, doi:10.1021/acsami.5b12588 (2016).

16 Pailler-Mattei, C., Bec, S. & Zahouani, H. In vivo measurements of the elastic mechanical properties of human skin by indentation tests. *Med Eng Phys* **30**, 599-606, doi:10.1016/j.medengphy.2007.06.011 (2008).

17 Liang, X. & Boppart, S. A. Biomechanical properties of in vivo human skin from dynamic optical coherence elastography. *IEEE Trans Biomed Eng* **57**, 953-959, doi:10.1109/TBME.2009.2033464 (2010).

18 Gong, S. *et al.* Tattoolike Polyaniline Microparticle-Doped Gold Nanowire Patches as Highly Durable Wearable Sensors. *ACS applied materials & interfaces* **7**, 19700-19708 (2015).

19 Yamada, T. *et al.* A stretchable carbon nanotube strain sensor for human-motion detection. *Nat Nanotechnol* **6**, 296-301, doi:10.1038/nnano.2011.36 (2011).

20 Amjadi, M., Pichitpajongkit, A., Lee, S., Ryu, S. & Park, I. Highly stretchable and sensitive strain sensor based on silver nanowire-elastomer nanocomposite. *ACS nano* **8**, 5154-5163, doi:10.1021/nn501204t (2014).

21 Trung, T. Q. & Lee, N. E. Flexible and Stretchable Physical Sensor Integrated Platforms for Wearable Human-Activity Monitoringand Personal Healthcare. *Advanced materials* **28**, 4338-4372, doi:10.1002/adma.201504244 (2016).

22 MacRitchie, J. & Bailey, N. J. Efficient Tracking of Pianists’ Finger Movements. *Journal of New Music Research* **42**, 79-95, doi:10.1080/09298215.2012.762529 (2013).

23 Suzuki, K. *et al.* Rapid-Response, Widely Stretchable Sensor of Aligned MWCNT/Elastomer Composites for Human Motion Detection. *ACS Sensors* **1**, 817-825, doi:10.1021/acssensors.6b00145 (2016).

24 Grimaldi, G. & Manto, M. Neurological tremor: sensors, signal processing and emerging applications. *Sensors (Basel)* **10**, 1399-1422, doi:10.3390/s100201399 (2010).

25 Massano, J. & Bhatia, K. P. Clinical approach to Parkinson's disease: features, diagnosis, and principles of management. *Cold Spring Harb Perspect Med* **2**, a008870, doi:10.1101/cshperspect.a008870 (2012).

26 Oh, S. J. *Clinical electromyography: nerve conduction studies*. (Lippincott Williams & Wilkins, Philadelphia, 2003).

27 Cupido, C. M., Galea, V. & McComas, A. J. Potentiation and depression of the M wave in human biceps brachii. *J Physiol* **491 ( Pt 2)**, 541-550, doi:10.1113/jphysiol.1996.sp021238 (1996).

28 Hufschmidt, A. Acoustic phenomena in the latent period of skeletal muscle: a simple method for in-vivo measurement of the electro-mechanic latency (EML). *Pflugers Arch* **404**, 162-165, doi:10.1007/BF00585413 (1985).

29 Sasaki, K., Sasaki, T. & Ishii, N. Acceleration and force reveal different mechanisms of electromechanical delay. *Med Sci Sports Exerc* **43**, 1200-1206, doi:10.1249/MSS.0b013e318209312c (2011).

30 Sandow, A. Studies on the latent period of muscular contraction. Method. General properties of latency relaxation. *Journal of Cellular and Comparative Physiology* **24**, 221-256, doi:10.1002/jcp.1030240306 (1944).

31 Haugen, P. & Sten-Knudsen, O. Sarcomere lengthening and tension drop in the latent period of isolated frog skeletal muscle fibers. *J Gen Physiol* **68**, 247-265, doi:10.1085/jgp.68.3.247 (1976).

32 Pan, F. *et al.* 3D graphene films enable simultaneously high sensitivity and large stretchability for strain sensors. *Advanced Functional Materials* **28**, 1803221 (2018).

33 Xu, M. *et al.* Stretchable and multifunctional strain sensors based on 3D graphene foams for active and adaptive tactile imaging. *Science China Materials* **62**, 555-565 (2019).

34 Liao, X. *et al.* Ultrasensitive and stretchable resistive strain sensors designed for wearable electronics. *Mater Horiz* **4**, 502-510, doi:10.1039/c7mh00071e (2017).

35 Everett, T. & Kell, C. *Human movement : An introductory text*. 6th ed. / edited by Tony Everett and Clare Kell. edn, (Elsevier health sciences, New York, 2010).

36 Cavanagh, P. R. & Komi, P. V. Electromechanical delay in human skeletal muscle under concentric and eccentric contractions. *Eur J Appl Physiol Occup Physiol* **42**, 159-163, doi:10.1007/BF00431022 (1979).

37 Norman, R. W. & Komi, P. V. Electromechanical delay in skeletal muscle under normal movement conditions. *Acta Physiol Scand* **106**, 241-248, doi:10.1111/j.1748-1716.1979.tb06394.x (1979).

38 Valencic, V. & Knez, N. Measuring of skeletal muscles' dynamic properties. *Artif Organs* **21**, 240-242, doi:10.1111/j.1525-1594.1997.tb04658.x (1997).

39 Ibitoye, M. O., Hamzaid, N. A., Zuniga, J. M. & Abdul Wahab, A. K. Mechanomyography and muscle function assessment: a review of current state and prospects. *Clin Biomech (Bristol, Avon)* **29**, 691-704, doi:10.1016/j.clinbiomech.2014.04.003 (2014).
